# Selective Laser Sintering and Graphitization for Mono‐Matrix Unibody Fabrication of Integrated Stereo‐Circuit and Stereo‐Fluidic Wearable Electronics

**DOI:** 10.1002/adma.75075

**Published:** 2026-09-21

**Authors:** Li Zhang, Wing Cheung Mak

**Affiliations:** ^1^ Department of Biomedical Engineering The Chinese University of Hong Kong Hong Kong China

**Keywords:** integrated bioelectronic‐fluidic platform, printed microfluidics, printed transducers, selective laser sintering and graphitization (SLSG), wearable biosensors

## Abstract

Wearable integrated electronic devices for biomedical, robotics, and sensing applications require greater compactness and functional complexity while maintaining wearability. However, seamlessly integrating electronic and microfluidic components remain challenging due to fabrication heterogeneity, material mismatches, and complex assembly requirements. In this study, we present an innovative selective laser sintering and graphitization (SLSG) 3D printing technology that enables seamless integration of 3D circuits (stereo‐circuits) and 3D fluidic channels (stereo‐fluidics). SLSG achieves unibody, autonomous mono‐matrix fabrication through adaptive photonic modulation of carbon‐rich precursors (e.g., polyetheretherketone (PEEK) powder), simultaneously creating insulating sintered structures and conductive graphitized patterns with conductivity up to 1,895 S/m, yielding 7.64‐fold higher electrochemical performance than classic glassy carbon electrodes (GCEs). Using SLSG, we fabricated a watch‐type wearable unibody multimodal microfluidic biosensor (UMMB) with integrated electrochemical biosensors for on‐body sweat metabolite monitoring. UMMB incorporates stereo‐circuits for sensing and signal routing, plus stereo‐fluidic channels for sweat respiration, achieving effective lactate and glucose sensing. The device demonstrates excellent sensitivities of 29.43 and 16.86 µA/mM for detecting lactate and glucose, respectively, while exhibiting nondetectable interference or circuit‐fluidic crosstalk. This novel SLSG 3D printing technology introduces a transformative unibody approach to fabricate multifunctional bioelectronic‐fluidic platform for integrated point‐of‐care devices, wearable sensors/biosensors, actuators, advanced theranostics, and personalized biomedical devices.

## Graphical abstract

A novel selective laser sintering and graphitization (SLSG) 3D printing technology enables unibody fabrication of compacted insulating structures and conductive circuits in fluidic‐integrated wearable electronics through adaptive photonic modulation. This autonomous mono‐matrix approach seamlessly integrates stereo‐circuits and stereo‐fluidics, demonstrated in a watch‐type unibody multimodal microfluidic biosensor (UMMB) for effective on‐body sweat metabolite monitoring.

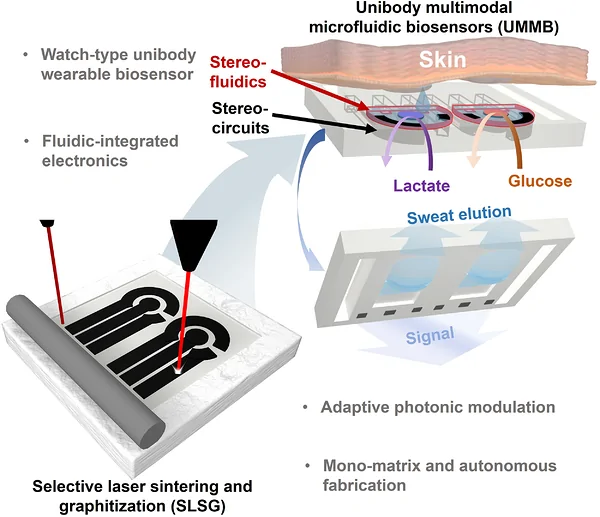

## 1 Introduction

Wearable integrated electronic devices are increasingly vital for biomedical applications, actuators/robotics, and sensors/biosensors, requiring greater compactness and functional complexity while maintaining their wearable characteristics [1, 2, 3]. A wide variety of wearable integrated electronic devices with physical sensors have been translated for commercialization, aimed at tracking body temperature [4], respiratory rate [5], and electrical signals including electroencephalography (EEG) [6], electromyography (EMG) [7], and electrocardiogram (ECG) [8]. Although these biophysical parameters provide valuable insights into general health information, they lack detailed information on dynamic biochemical and metabolic processes related to physiological status [9]. In contrast, electrochemical biosensors employ intricate transducer configurations (e.g., bioreceptor‐to‐transducer interfaces, transducer signal routing) [10] to noninvasively measure biofluids like saliva, sweat, tears, and interstitial fluids, offering the potential to radically improve health status assessment by tracking changes in metabolic processes [11]. Among these, sweat is particularly attractive due to its ease of collection and rich source of biomarkers (e.g., electrolytes, metabolites, proteins, cytokines) related to important health conditions such as dehydration, physical fatigue, mental stress, and disease [12]. The inherent complexity of sweat biomarkers creates a need for multimodal biosensing platforms capable of providing more comprehensive information about our bodily status [13]. To achieve effective biofluidic detection, microfluidics is essential in biosensing for sweat sample transport, providing reaction chambers for initiating bioreactions, and channels for delivering sweat samples to the transducer interface [14]. Consequently, there are pressing demands for the integration of electronics/sensors and microfluidics, while the multimodal biosensing complexity requires more advanced fabrication and integration methods. Nevertheless, current fabrication and integration technologies face unmet challenges, including functional challenges arising from post‐integration or material modification (e.g., circuit‐fluidic crosstalk) and the geometric complexities of bonding, assembly, and alignment steps inherent to conventional multi‐material or multi‐method approaches.

Existing fabrication technologies for transducer components include several established methods, such as screen printing [15], inkjet printing [16], and laser direct writing [17, 18]. Screen printing, which requires tailor‐designed masks to guide the deposition of conductive inks into precise electrode patterns, has become widely adopted due to its capability for high‐throughput fabrication of printed electrodes [15]. While this technique suffers from inflexibility when electrode design changes are needed, necessitating the time‐consuming and costly redesign and fabrication of new masks. Alternatively, inkjet printing offers enhanced flexibility in design and fabrication of printed electrodes, though it demands specialized ink formulation to prevent nozzle clogging during the printing process and requires specific solvent systems that support rapid evaporation and drying [16]. More recently, laser direct writing has emerged as a powerful technique that enables precise patterning of conductive materials through photothermal or photochemical processes, offering the advantages of mask‐free and ink‐free fabrication [17, 18]. However, it struggles to create complex multilayer architectures without multiple processing steps. Microfluidic fabrication technology employs diverse conventional approaches like photolithography, [19] computer numerical control (CNC) micromilling and laser cutting, [20] semisolid extrusion (SSE), [21] etc. Photolithography enables precise and high‐resolution fabrication of microfluidic devices using materials like polydimethylsiloxane (PDMS), yet requires labor‐intensive fabrication steps and specially equipped light‐controlled environments [19]. CNC micromilling and laser cutting prove effective for manufacturing fluidic devices with rigid materials such as glass, silicon, and various polymers including cyclic olefin copolymer (COC) [22] and polymethyl methacrylate (PMMA) [23], though these processes may create undesirable surface defects when applied to temperature‐sensitive, thin, or fragile substrates [20]. Meanwhile, SSE has gained attention for its ability to directly print microfluidic channels through layer‐by‐layer deposition of viscous materials, offering rapid prototyping capabilities without requiring molds or masks [21]. However, the building material requires specialized rheological properties (specific viscosity range and shear‐thinning behavior), and switching between materials with different properties during printing remains challenging.

Despite development in technologies for fabricating electronic transducers and microfluidic components, integration technologies pose considerable challenges. Several research groups have attempted to address these issues. For example, Song et al. demonstrated a combination of SSE with layer assembly to fabricate multimodal sensors for measuring glucose, alcohol, and pH in sweat along with heart rate and temperature, though their approach still requires precise layer alignment during assembly [21]. Yang et al. combined laser direct writing, laser cutting, and double‐sided tape adhesion to create integrated microfluidic sensors for simultaneous measurement of uric acid and tyrosine, yet the multi‐step process remains complicated and alignment‐dependent [24]. Xu et al. integrated inkjet printing with laser cutting and plasma‐treated PDMS to fabricate multimodal microfluidic sensors for monitoring key stress‐related physiological signals (pulse waveform, galvanic skin response, and skin temperature) and sweat metabolites (glucose, lactate, and uric acid) and electrolytes (Na^+^, K^+^, and NH_4_^+^) during daily activities. However, their method necessitates cleanroom facilities, precise layer assembly, and skilled personnel [25]. These recent approaches to integrating transducer and microfluidic components have yielded notable advances, though considerable limitations persist, including dependence on cleanroom infrastructure, multiple fabrication steps, layer‐by‐layer assembly requirements, alignment procedures, and the inability to achieve seamless integration. The fundamental challenges of seamless integration stem from functional complexity requirements, material mismatches, and technical heterogeneity across fabrication methods. Therefore, there remains a pressing need for an advanced fabrication method that can achieve seamless and effective fabrication and integration with mono‐matrix capability (single material during fabrication and integration) to overcome these challenges.

Nowadays, 3D printing technologies offer promising capabilities for creating complex 3D structures in single fabrication processes that can overcome alignment and assembly limitations. Several pioneering efforts have been made to build the foundation for seamlessly integrated electronic devices. Wu et al. demonstrated a 3D wireless passive sensor using FDM multiple‐nozzle printing with silver particle metallic embedded interconnects [26], while Lopes et al. relied on a two‐step process, fabricating 3D structures by stereolithography (SLA) followed by post‐fabrication circuit integration via direct ink writing (DIW) [27]. Xiao et al. employed a dual‐light curing process to fabricate 3D embedded electronics using polyethylene glycol diacrylate (PEGDA) loaded with various concentrations of AgNO_3_ [28]. Despite these advances, current attempts still suffer from multiple fabrication steps, destructive stops between printing phases, frequent material or method changes, technical heterogeneity, and complicated post‐processing requirements. These limitations underscore the need for a new 3D printing method that can achieve mono‐matrix fabrication for seamless integration.

In this work, we introduce selective laser sintering and graphitization (SLSG), an innovative 3D printing approach based on unibody design that enables effective, seamless, and mono‐matrix fabrication and integration of wearable microfluidic‐integrated biosensors, as shown in Scheme 1. Unibody design refers to a manufacturing approach where a product's structure is created as a single, integrated piece rather than assembled from multiple separate components. This concept has had revolutionary impacts on the automotive, aerospace, consumer electronics, and medical device industries [29], and is now impacting the wearable microfluidic‐integrated biosensor field. The significance of this unibody design for microfluidic‐integrated biosensors lies in its capability to simultaneously fabricate both microfluidics and electrodes within a single process. SLSG employs unibody design for seamless and effective fabrication and integration of microfluidics and electrodes by simultaneously fabricating both insulating structures and conductive patterns via laser power control. Moreover, the SLSG is capable of mono‐matrix fabrication by utilizing a carbon‐rich powder precursor (e.g., polyetheretherketone (PEEK)) that can be converted into both insulating structures and conductive patterns. The unique mechanism of SLSG lies in adaptive photonic modulation: a relatively lower laser power induces the physical fusion of precursor particles, creating sintered structures through densification between adjacent particles, while a relatively higher laser power induces the graphitization of carbon‐rich precursor materials into conductive graphitic structures through a photothermal chemical process, creating patterned electrical circuits and electrodes with good conductivity. Leveraging the advantages of 3D printing, SLSG enables the fabrication of three‐dimensional (3D) circuits (stereo‐circuits) and 3D fluidic channels (stereo‐fluidics) in a unibody design, thereby facilitating the creation of personalized, wearable, and integrated electronic devices.

**SCHEME 1:**
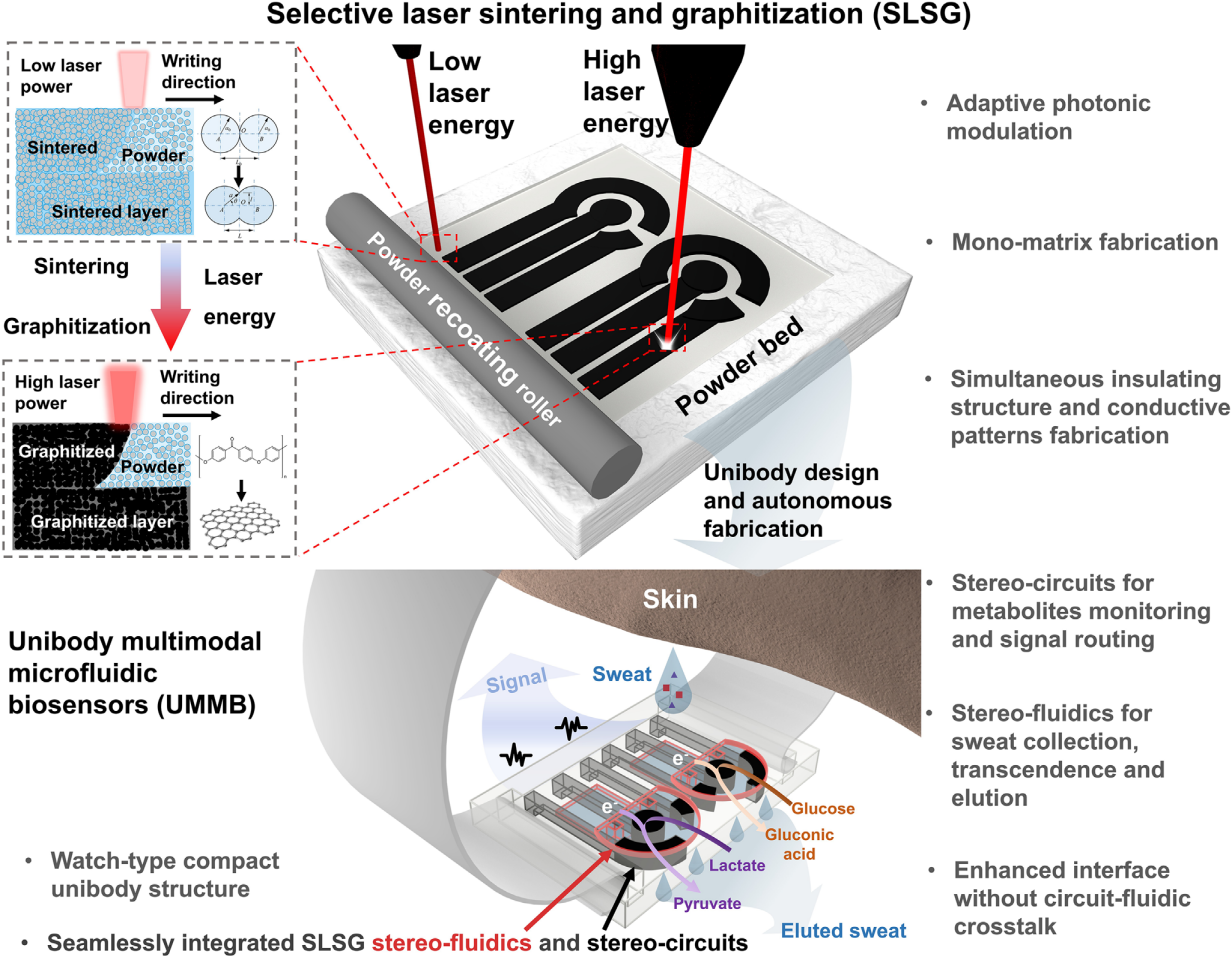
Schematic diagrams of the SLSG process and the watch‐type UMMB for metabolites monitoring in sweat.

To demonstrate the practical applications of SLSG, we have developed a watch‐type unibody multimodal microfluidic biosensor (UMMB) device from the PEEK powder precursor, featuring seamlessly integrated stereo‐circuit and stereo‐fluidic components (Scheme 1). The stereo‐circuit in UMMB incorporates an array of electrochemical biosensors capable of detecting lactate and glucose simultaneously for multimodal metabolite monitoring and efficient signal routing. The stereo‐fluidic channels enable sweat collection, transcendence, and elution for sweat respiration, providing circuit‐fluidic interfaces without circuit‐fluidic crosstalk. To optimize UMMB performance, we conducted systematic SLSG parameter optimization, stereo‐circuit and stereo‐fluidic demonstration, material and morphological characterization, and electrochemical characterization. The UMMB serves as a compelling demonstration of SLSG's capacity to address core challenges in fabricating microfluidic‐integrated wearable electronic devices, offering a transformative unibody design approach for multifunctional and multimodal biomedical devices, actuators, and wearable sensors/biosensors.

## 2 Result and Discussion

### 2.1 SLSG Process and Printed Stereo‐Circuits and Stereo‐Fluidics

Our SLSG process enables mono‐matrix fabrication to allow simultaneous sintering and graphitization by adaptive photonic modulation. PEEK powder is a thermoplastic polymer that is able to undergo sintering where particle necking and interparticle fusion happen between particles at a relatively low localized temperature under its melting point (∼343°C), induced by the applied laser energy [30, 31, 32, 33, 34, 35, 36]. Besides, as a carbon‐rich precursor, the abundant phenyl groups in its molecular structure provide its ability of local atom rearrangement and photothermal graphitization to be converted to graphite‐like material under a relatively high localized temperature (>∼2000°C) induced by higher laser energy [37]. These unique properties of PEEK enables the laser‐parameter‐controlled simultaneous SLSG mono‐matrix fabrication of geometry‐defined functional components. We investigated several key laser parameters such as laser power, laser scan speed and laser defocus distance [38] to reveal the transition of sintering and graphitization of PEEK and modulate the insulating structure and conducting patterns.

Figure 1a‐i shows the optical images of SLSG objects (1 cm*1 cm) fabricated under different laser power (*x*‐axis, varied from 2.5 to 6.8 W) and laser defocus distance (*y*‐axis, varied from 0 to 10 mm) with the laser scan speed of 100 mm/s in the form of a heat map. The results show that the surface color of SLSG objects transitions gradually from white (the color of PEEK powder), indicating sintering, to relatively darker colors with increasing laser power, indicating progressive graphitization. The results were analyzed using grayscale values of the SLSG object surfaces to reflect the level of graphitization (Figure S1) [39]. Objects with grayscale values below 50% were classified as sintered SLSG objects, those with values between 50% and 90% were considered partially graphitized, and those with values above 90% were classified as graphitized SLSG objects. The boundaries between sintered, partially graphitized, and graphitized SLSG objects are indicated by red and blue lines, respectively, demonstrating the synergistic relationship between laser power and defocus distance in modulating the sintering‐to‐graphitization transition in the SLSG process. Furthermore, electrical resistance measurements of the SLSG objects using an ohmmeter (Figure S2a) revealed that the sintered and partially graphitized objects are relatively insulating with resistances exceeding 1 kΩ, while the graphitized objects exhibit resistances below 1 kΩ and sufficient conductivity to illuminate an LED, demonstrating their electrical functionality (Figure S2b).

**FIGURE 1:**
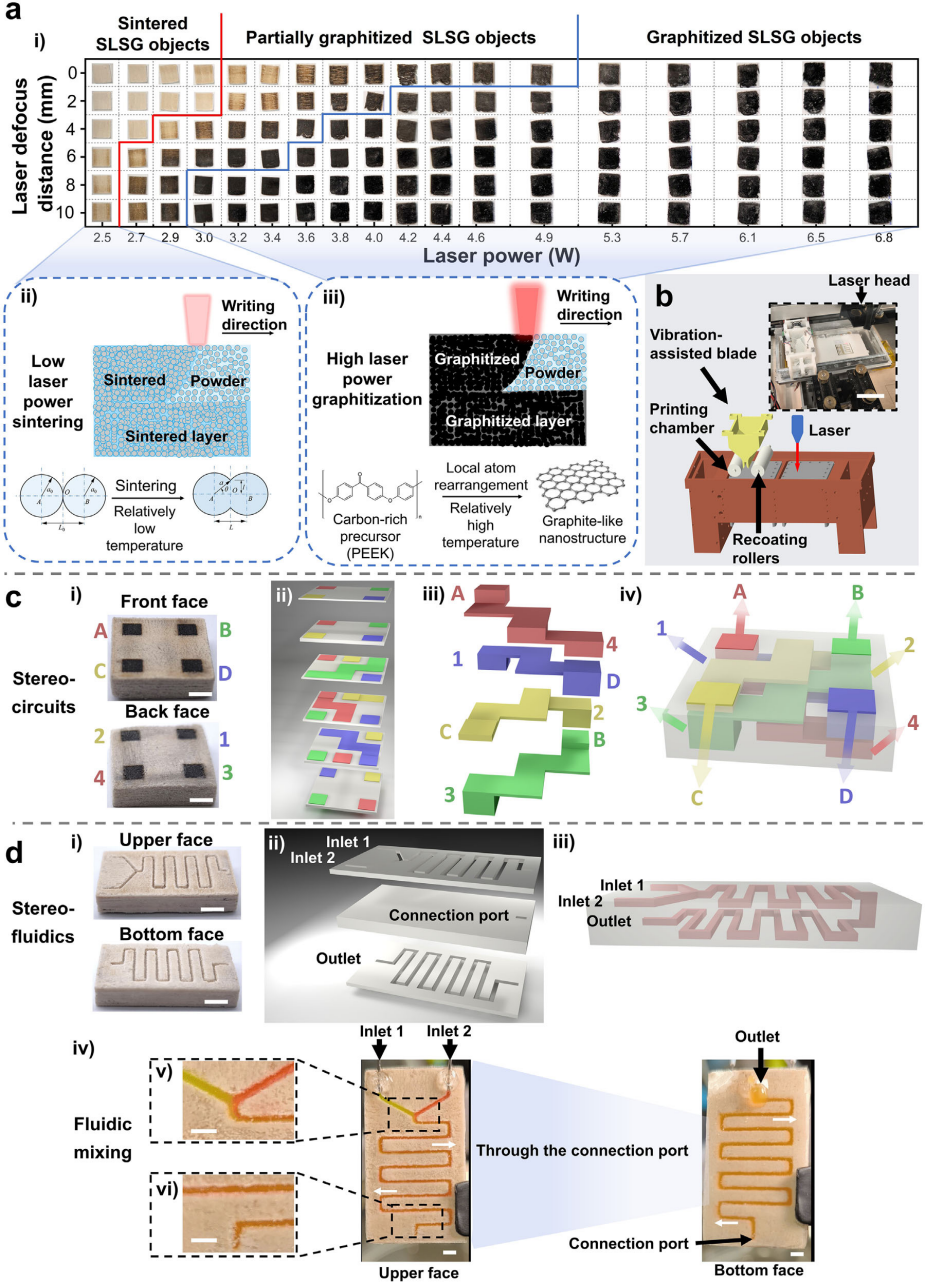
SLSG process and printed stereo‐circuits and stereo‐fluidics. (a) SLSG objects fabricated under different parameters visualized as a heatmap (i) with schematic diagrams of the sintering mechanism (ii) and graphitization mechanism (iii). (b) Schematic diagram of the SLSG fabrication platform, inset shows a photograph of the SLSG platform, scale bar: 44 mm. (c) A SLSG printed stereo‐circuit hub (i, scale bar: 4.5 mm) and its schematic layered view (ii), exploded view (iii) and perspective view (iv). (d) A SLSG‐printed stereo‐fluidic device (i, scale bar: 5.7 mm) and its schematic layered view (ii) and perspective view (iii); (iv) fluidic mixing test of the stereo‐fluidic device showing characteristic of laminar flows (v) before mixing and the orange color after mixing (vi), scale bar: 2.9 mm, white arrows indicate the flow direction.

The advanced mono‐matrix simultaneous laser power‐induced transition of sintering and graphitization lies in the unique mechanism of SLSG process. As shown in Figure 1a‐i), during sintering, thermoplastic PEEK polymer powder forms necks between particles through atomic diffusion via solid‐state sintering (SSS). SSS occurs at relatively low applied laser energies, where PEEK particles are heated below a threshold temperature, typically their melting point (∼343°C), to prevent polymer degradation [40, 41]. However, as illustrated in Figure 1a‐iii, when the applied laser energy exceeds a threshold, the generated high localized temperatures (>∼2000°C) is able to initiate local atom rearrangement of the carbon‐rich PEEK precursor to drive *sp^3^*‐to‐*sp^2^* carbon hybridization, so called the photothermal graphitization, forming graphite‐like material with high electric conductivity [42].

The laser defocus distance of the CO_2_ laser with Gaussian intensity distribution governs the level of graphitization. Larger defocus distances result in larger laser spot sizes and thus more overlap during laser scanning (detailed transformation shown in the Supporting Information) [43]. Increased scanning overlap compensates for reduced laser power, explaining why graphitization under defocused conditions occurs at lower laser powers. This adaptive photonic modulation of sintering and graphitization lays the foundation of the simultaneous fabrication of insulating structures and conductive patterns, enabling the integration of stereo‐circuits and stereo‐fluidics.

To demonstrate SLSG's capability for building complex structures such as stereo‐circuits and stereo‐fluidics, we developed an SLSG fabrication platform integrated with a CO_2_ laser, as shown in Figure 1b (inset shows a photograph of the SLSG platform). The system features a custom‐built automated feeding system comprising a printing chamber, a vibration‐assisted blade, and counter‐rotating rollers. The stereo‐circuits and stereo‐fluidics employ a unibody design approach using computer‐aided design (CAD), which is subsequently converted to G‐code and uploaded to the SLSG printing platform. The autonomous SLSG printing process then commences with the blade coating the powder bed, followed by the CO_2_ laser head writing according to set laser parameters and designed patterns to achieve selective sintering and graphitization. Upon completion of each layer, the printing chamber descends by a specified layer thickness before the blade recoats the subsequent layer. This process repeats to obtain the final SLSG‐printed objects (details in Supporting Information).

Figure 1c‐i presents an SLSG‐printed stereo‐circuit hub (20×20×5 mm) featuring eight surface electrodes (A, B, C, D on the front face; 1, 2, 3, 4 on the back face) connected counterintuitively through diagonal internal pathways. The hub was printed using parameters of 100 mm/s speed, 2.9 W power, and 0 mm defocus distance for sintering, and 100 mm/s speed, 5.7 W power, and 6 mm defocus distance for graphitization. The stereo‐circuits hub functions with the diagonal internal connections shown in its schematic layered view (Figure 1c‐ii), exploded view (Figure 1c‐iii) and perspective view (Figure 1c‐iv). To test the stereo‐circuits hub, we designed and developed a test bench (Figure S3a) that connected and wired the eight electrodes (Figure S3b) to two terminals with four channels for testing (Figure S3c). An LED was connected to individual channels between the two terminals to verify connectivity (Figure S3d). Results confirmed that only the complementary electrode pairs (A→4, D→1, C→2, and B→3) successfully illuminated the LEDs, while noncomplementary connections did not light up, verifying the effectiveness of the stereo‐circuits without crosstalk.

Figure 1d‐i showcases an SLSG‐printed stereo‐fluidic device (35×20×6 mm) featuring serpentine mixing channels (1×1 mm) on both upper and bottom faces, printed at 100 mm/s speed, 2.9 W power, and 0 mm defocus distance. As illustrated in the schematic layered view (Figure 1d‐ii) and perspective view (Figure 1d‐iii), the stereo‐fluidic device comprises two inlets on the upper face, one outlet on the bottom face, and two serpentine channels on both surfaces connected through a connection port. Figure 1d‐iv demonstrates the fluid mixing test, where yellow and red dye solutions were loaded through the two inlets at 0.5 mL/min. Prior to entering the serpentine channel, the dye solutions exhibited the characteristic of laminar flows (Figure 1d‐v) [44]. After traversing the serpentine channel on the upper face, the orange color emerged due to chaotic advection of the yellow and red dyes (Figure 1d‐vi). The dye solutions were then directed through the connection port from the upper face before being eluted from the bottom face. To quantitatively analyze the mixing performance, the hue (H) values in the HSB color model were extracted at representative positions along the stereo‐fluidic channel (Figure S4). The H value encodes the dominant color of the fluid as an angular coordinate (in degrees) and therefore serves as a direct optical indicator of the local mixing performance of the two colored streams [45]. The two inlet streams showed distinct H values of 51.7° (yellow, inlet 1) and 16.3° (red, inlet 2), which progressively converged to intermediate orange values of 31.3° at the upper‐face connection port, 35.7° at the bottom‐face connection port, and 39.0° at the outlet. This convergence of the H values confirms effective mixing of the two streams as they traverse the upper and bottom serpentine channels through the 3D connection port. The unique stereo‐fluidic feature enables multi‐dimensional fluid manipulation and emulation of biological environments in compact devices (e.g., point‐of‐care diagnostics, organ‐on‐a‐chip systems, and wearable devices) [46, 47]. Additionally, no observable leakage occurred during flow testing, confirming the effectiveness of the SLSG stereo‐fluidics. Owing to the millimeter‐scale channel dimensions (1×1 mm) and the low‐Reynolds‐number laminar flow regime relevant to sweat transport, the influence of the printed surface roughness on fluid transport is negligible relative to channel geometry and driving pressure [48]. These results demonstrate SLSG's capability to simultaneously print insulating structures and conductive patterns for integrating functional stereo‐circuits and stereo‐fluidics through adaptive photonic modulation of sintering and graphitization using mono‐matrix materials.

### 2.2 Optimization and Characterization of SLSG Process

Optimization of laser parameters was carried out to establish the working conditions for the SLSG process. Quantitative analysis of the conductivity of graphitized objects fabricated under different laser powers (*x*‐axis, varying from 3.04 to 6.84 W) and laser defocus distances (*y*‐axis, varying from 0 to 10 mm) was conducted using the four‐probe technique [49], as shown in Figure 2a. The results show that conductivity initially increases and then decreases with increasing laser power [50]. Under 6 mm defocus distance, graphitization at a laser power of 5.70 W and 6 mm defocus distance achieved the highest conductivity of 1895 S/m, surpassing that of classic laser‐induced graphitization on polyimide (PI) [51]. Therefore, the optimal graphitization parameters were determined to be 5.70 W laser power and 6 mm defocus distance. Optimization of sintering parameters was conducted as shown in Figure 1a‐i. At 2.90 W laser power and 0 mm defocus distance, the surface of the sintered object remained intact without observable cracks and remained a predominantly white color, indicating effective sintering. Consequently, the optimal sintering parameters were set as 2.90 W laser power and 0 mm defocus distance.

**FIGURE 2:**
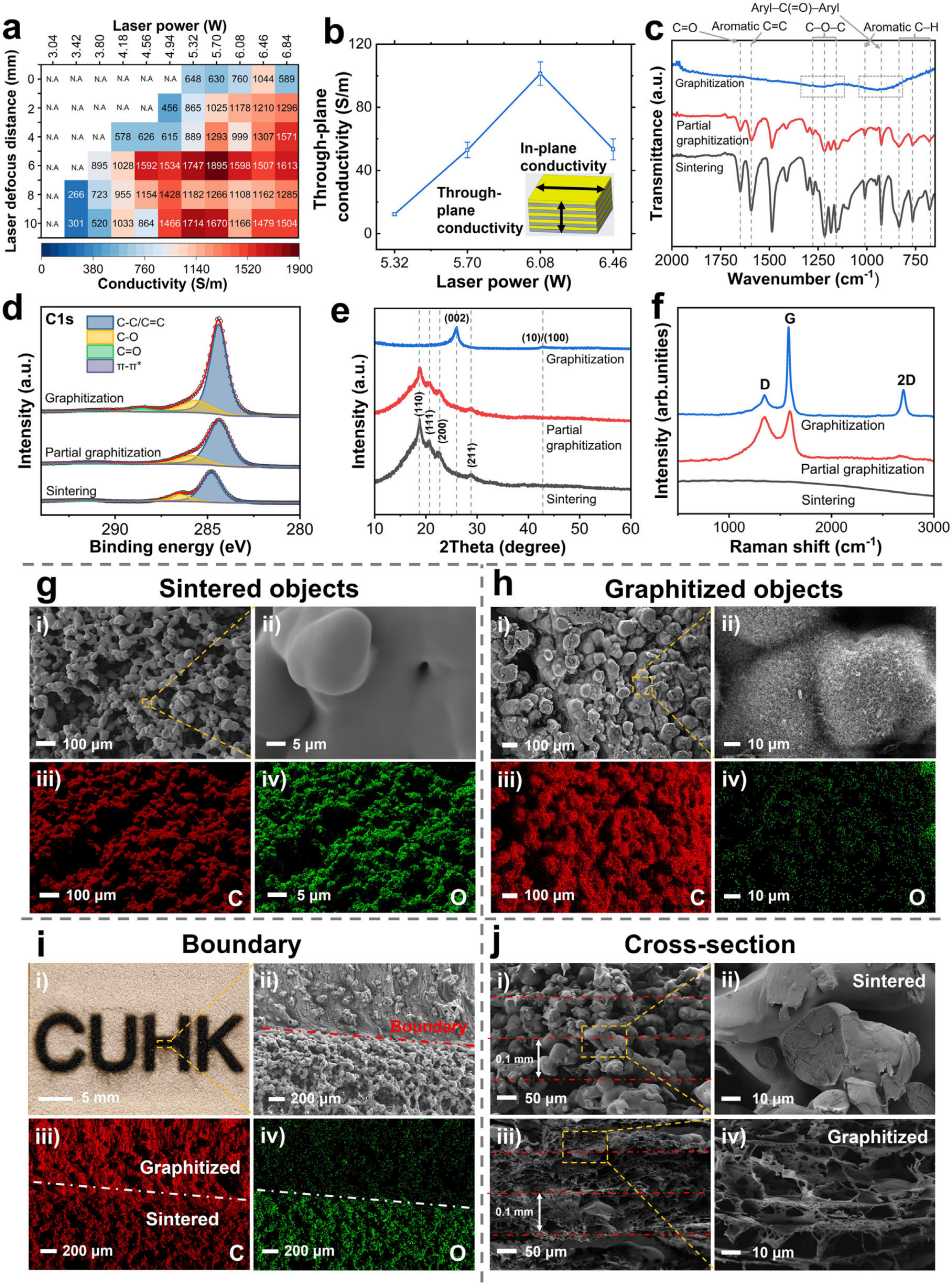
SLSG optimization and characterization. (a) In‐plane conductivity of SLSG objects fabricated under different parameters visualized as a heatmap. Values in the heat map are in the unit of S/m, the “N.A” means conductivity not detectable. (b) Through‐plane conductivity of the SLSG graphitized objects under different laser powers. (c) FTIR, (d) high‐resolution C1s XPS, (e) XRD and (f) Raman spectra of sintered, partially graphitized and graphitized samples. (g) SEM image (i) and high‐magnification SEM image (ii) of the sintered objects with corresponding EDS mapping of C (iii) and O (iv). (h) SEM image (i) and high‐magnification SEM image (ii) of the graphitized objects with corresponding EDS mapping of C (iii) and O (iv). (i) A SLSG object with a “CUHK” pattern created by laser writing (i) and the SEM image of the boundary between sintering and graphitization (ii) with corresponding EDS mapping of C (iii) and O (iv). (j) Cross‐section SEM images of the sintered (i) with magnified view (ii) and graphitized objects (iii) with magnified view (iv).

Optimization of the layer feeding thickness for SLSG is presented in Figure S5, which shows optical images of sintered objects (7.5×7.5×2.5 mm cubes) fabricated with different feeding layer thicknesses ranging from 0.1 to 0.3 mm. The results demonstrate that with increasing feeding layer thickness, delamination occurs due to lower distributed laser energy per unit volume of powder particles. At 0.1 mm feeding layer thickness, the minimum thickness achievable by the powder feeding platform, the sintered object remains intact, indicating effective interlayer bonding with sufficient resolution and structural complexity for unibody device fabrication. Therefore, the feeding layer thickness was determined to be 0.1 mm for fabricating 3D SLSG objects. To further validate the object thickness accuracy, the thickness of SLSG objects printed at the 0.1 mm feeding layer thickness was measured using a thickness gauge (precision 0.01 mm, Figure S7). For sintered and graphitized objects designed with 25 layers at a nominal 0.1 mm layer thickness (designed object thickness 2.5 mm), the sintered and graphitized objects exhibited measured thicknesses of 2.53 ± 0.04 mm and 2.50 ± 0.02 mm, corresponding to effective layer thicknesses of 0.101 and 0.100 mm and maximum object thickness deviations of only ∼1.6% and ∼0.8% from the designed values, respectively, confirming good layer‐thickness accuracy and consistent interlayer bonding with acceptable tolerance compared to common desktop (e.g. ± 0.2 mm for desktop FDM 3D printer) and commercial polymer additive‐manufacturing systems (e.g. ± 0.05 mm for commercial SLA 3D printer) [52]. Quantitative analysis of the through‐plane conductivities of 3D graphitized SLSG objects under different laser powers is shown in Figure 2b, with corresponding optical images presented in Figure S6. The through‐plane conductivity initially increases and then decreases with increasing laser power, ranging from 5.32 to 6.46 W, achieving a maximum through‐plane conductivity of approximately 101 S/m at 6.08 W. The initial rise reflects increasing graphitization and the formation of ordered sp^2^‐rich conductive networks with rising laser energy, whereas beyond the laser power of 6.08 W, excessive laser energy induces thermal ablation and local fragmentation of the graphitized network, degrading the vertical conductive pathways and lowering the through‐plane conductivity [53]. Notably, the highest through‐plane conductivity occurs at 6.08 W laser power, which exceeds the 5.70 W optimized for graphitization, because a portion of the laser power is distributed to interlayer bonding. For electrochemical applications, the in‐plane conductivity and electroactivity of the outermost electrode surface are critical on the device performance. Thus, 5.70 W was selected as the laser power for 3D SLSG object graphitization.

The sintered, partially graphitized, and graphitized samples were characterized by Fourier transform infrared spectroscopy (FTIR), x‐ray photoelectron spectroscopy (XPS), x‐ray diffraction (XRD), and Raman spectroscopy. FTIR was performed to track the chemical‐bond evolution accompanying the sintering‐to‐graphitization transition. In the FTIR spectra (Figure 2c), the sintered sample (processed at the optimal 3.42 W and 6 mm defocus) exhibited the characteristic PEEK absorption bands, including diaryl ketone C═O stretching at ∼1649 cm^−1^, aromatic C═C ring skeletal vibrations at ∼1593 and ∼1487 cm^−1^, aryl ether C─O─C vibrations in the 1277–1157 cm^−1^ region, the Aryl–C(═O)–Aryl fingerprint band at ∼926 cm^−1^, and aromatic C─H vibrations in the 1010–674 cm^−1^ region [54]. The preservation of these characteristic bands indicates the chemical structure of PEEK was retained during the laser sintering process [40, 41]. In comparison, the partially graphitized sample (processed at 3.42 W and 6 mm defocus) displayed weakened PEEK adsorption bands, suggesting photothermal pyrolysis/destruction of PEEK functional groups driven by the photothermal reaction induced by relatively higher laser energy [37]. By contrast, the graphitized sample (processed at the optimized 5.70 W and 6 mm defocus), showed near‐complete disappearance of the characteristic PEEK absorption bands associate with C═O, aromatic C═C and aromatic C─H vibrations, while only two broad bands observed at ∼1277–1157 cm^−1^ and ∼1009–836 cm^−1^ corresponding to residual oxygen‐containing groups. This could be explained by the more intense chemical bond cleavage and re‐organization of the aromatic backbone into a graphite‐like structure via laser‐induced photothermal reaction [42].

XPS further verified the chemical evolution of the sintering and graphitization processes (Figures 2d and S8). The survey spectra show the sintered, partially graphitized, and graphitized samples are primarily composed of C and O, showing dominant C 1s (285 eV) and O 1s (532 eV) signals (Figure S8a). The C/O atomic ratio increases from 4.8 for the sintered sample to 7.9 for the partially graphitized sample, and further to 12.8 for the graphitized sample (Figure S8b), indicating progressive carbon enrichment and loss of oxygen driven by laser‐induced photothermal reaction [42]. High‐resolution C 1s spectra were deconvoluted into C─C/C═C, C─O, C═O, and π–π* shake‐up components (Figure 2d). The sintered sample showed a C─C/C═C‐dominated envelope (77.92%) with C─O (18.00%), C═O (0.03%), and π–π* (4.04%) contributions, consistent with PEEK object surfaces [55]. Partial graphitization retained dominant C─C/C═C (76.78%) with slightly increased C─O (18.43%), C═O (0.64%), and π–π* (4.15%), suggesting the onset of graphitization and surface oxidation induced by elevated laser energy [56]. For the envelope of graphitized sample, C─C/C═C remained dominant (74.77%) accompanied by C─O (17.91%) and π–π* (2.47%) contributions, whereas the increased C═O contribution (4.84%) may arise from residual oxygenated edge/defect sites within the graphitized network [57]. The XPS results indicate that laser graphitization induces cleavage of oxygen‐containing PEEK bonds, removal of oxygen‐containing groups, and reconstruction of the polymer backbone into a carbon‐rich, defective, and partially oxidized conjugated carbon network [56].

XRD analysis further elucidated the structural evolution of PEEK during SLSG (Figure 2e). The sintered sample retained the characteristic semicrystalline PEEK reflections at ∼18.7°, 20.7°, 22.7°, and 28.8°, corresponding to the (110), (111), (200), and (211) planes, respectively [58]. For the partially graphitized sample, these reflections became weaker and broader, suggesting progressive disruption of the semicrystalline PEEK lattice [59]. In contrast, the graphitized sample showed disappearance of the PEEK crystalline reflections and emergence of a dominant reflection at ∼26.0° and a broad reflection at ∼42.8°, assigned to graphitic carbon (002) interlayer stacking and in‐plane (10)/(100) ordering, respectively [60]. The broad graphitic reflections indicate defect‐rich, turbostratic graphene‐like structure rather than highly crystalline graphite [61].

Raman spectroscopy provided further insight into the evolution of laser‐power‐dependent graphitization process (Figures 2f and S9). In contrast to sintered samples, which exhibited no obvious bands, three characteristic bands appeared for partially graphitized samples including a D band at 1324 cm^−1^ associated with structurally disordered graphene, a G band at 1577 cm^−1^ corresponding to the *E_2g_* mode at the Γ‐point arising from stretching of a sp^2^ C─C bond in graphitic materials, and a 2D band at 2636 cm^−1^ indicated the stacking order of graphene layers and the initial formation of sp^2^ carbon domains (Figure 2f). For the graphitized sample, well‐defined D, G, and 2D bands further verified the conversion of carbon‐rich PEEK into graphitic/sp^2^ carbon structures [62, 63]. Quantitatively, as laser power increased from 3.42 to 5.70 W at the optimized 6 mm defocus distance, the I_D_/I_G_ ratio decreased from 0.89 to 0.22, while the calculated L_a_ increased from 21.69 to 87.59 nm, indicating progressive growth and ordering of sp^2^ domains with reduced defect density (Figure S9) [63]. L_a_ denotes the average in‐plane crystallite size of the graphitic domains and serves as a quantitative descriptor of graphitization quality, where a larger L_a_ reflects larger and more ordered sp^2^ domains with a lower defect density [64]. Further increasing laser power led to an increase in I_D_/I_G_ and a decrease in L_a_, suggesting the introduction of additional defects and over‐graphitization caused by excessive laser energy [65]. This result supports the mechanistic explanation of the increase and subsequent decrease of the in‐plane (Figure 2a) and through plane conductivity (Figure 2b) [53]. The I_2D_/I_G_ value of the optimized graphitized sample (∼0.30) is consistent with the characteristics of multilayer/turbostratic graphitic carbon, [66] and its lower I_D_/I_G_ and larger L_a_ than classic laser‐induced graphitized PI (Table S2) indicate a lower degree of disorder, resulting in better graphitization quality, which likely contributes to the higher conductivity of SLSG graphitized PEEK [53].

The surface morphologies of sintered and graphitized SLSG objects were examined using scanning electron microscopy (SEM). Figure 2g‐i, ii show top‐view and high‐magnification SEM images of the sintered object, respectively, revealing that adjacent PEEK powder particles form necks under laser energy to create an interconnected structure, supporting the sintering mechanism [40, 41]. In contrast, SEM images of the graphitized object display interconnected “fluffy” nanostructures, likely resulting from rapidly liberated gases produced by high‐temperature and photothermal reactions induced by relatively higher laser energy during graphitization (Figure 2hi, ii) [67]. Figure 2g‐iii, iv present corresponding energy‐dispersive x‐ray spectroscopy (EDS) mapping of the sintered object, showing 94% C and 6.0% O in atomic fraction, while the graphitized object exhibits 97.9% C and 2.1% O content in atomic fraction (Figure 2h‐iii, iv). The increased carbon and decreased oxygen content in the graphitized object result from the high‐temperature and photothermal reaction of PEEK, where rearrangement of carbonyl groups in the PEEK structure produces and releases oxygen‐containing gas molecules (e.g., CO, H_2_O), leading to the carbon enrichment and loss of oxygen, consistent with the FTIR and XPS results [68].

Figure 2i‐i displays a SLSG object with a “CUHK” pattern created by laser writing, demonstrating the ability of SLSG to produce arbitrary and customized patterns. A clear boundary between sintered and graphitized regions is visible in the SEM image of the SLSG object (Figure 2i‐ii), revealing distinct differences in surface morphology between the sintering and graphitization processes, which is further confirmed by corresponding EDS mapping (Figure 2i‐iii and 2i(iv)). Cross‐sectional SEM images of the 3D sintered object are presented in Figure 2j‐i, ii. The cross‐section of the sintered object displays an interconnected structure where necks form between particles of adjacent layers, creating interlayer bonding, supporting the sintering mechanism [40, 41]. Figure 2j‐iii, iv show cross‐sections of the 3D graphitized object, revealing an interconnected multi‐layer graphite flake structure resembling stacked cardboard‐like architecture. This interconnected layered structure contributes to through‐plane conductivity, with cavities between the interconnected graphite flake‐planes resulting from gas release during graphitization [68].

### 2.3 SLSG printed UMMB for metabolites detection and on‐body biosensing

The electrochemical properties of the SLSG electrode (Figure 3a inset) were investigated using cyclic voltammetry (CV) and electrochemical impedance spectroscopy (EIS) and compared to those of a classic glassy carbon electrode (GCE). Figure 3a shows the reversible redox peaks of the Fe(CN)_6_^3−^/^4−^ probe at the SLSG electrode, exhibiting an anodic peak current of 320.2 µA, representing a 7.64‐fold increase compared to the GCE (41.92 µA), and a lower onset potential of 0.04 V compared to 0.1 V for the GCE. The electroactive area (A) of the SLSG electrode was obtained from the plot of peak currents versus the square root of the scan rate according to the Randles–Sevcik equation (Figure S10) [69]. Using an apparent diffusion coefficient of Fe(CN)_6_^3−/4−^ of 7.6*10^−6^ cm^2^/s [70], the *A* value of the SLSG electrode was calculated to be 0.529 cm^2^ which is nearly 7.4 times larger than the geometric area (*A_geo_*) of the SLSG electrode (0.071 m^2^). This large active surface area and superior electrocatalytic activity of the SLSG electrode contribute to the enhanced electrode kinetics due to the interconnected “fluffy” nanostructures (Figure 2h‐ii) and the multilayer graphite flake structure (Figure 2j‐iv) [71].

**FIGURE 3:**
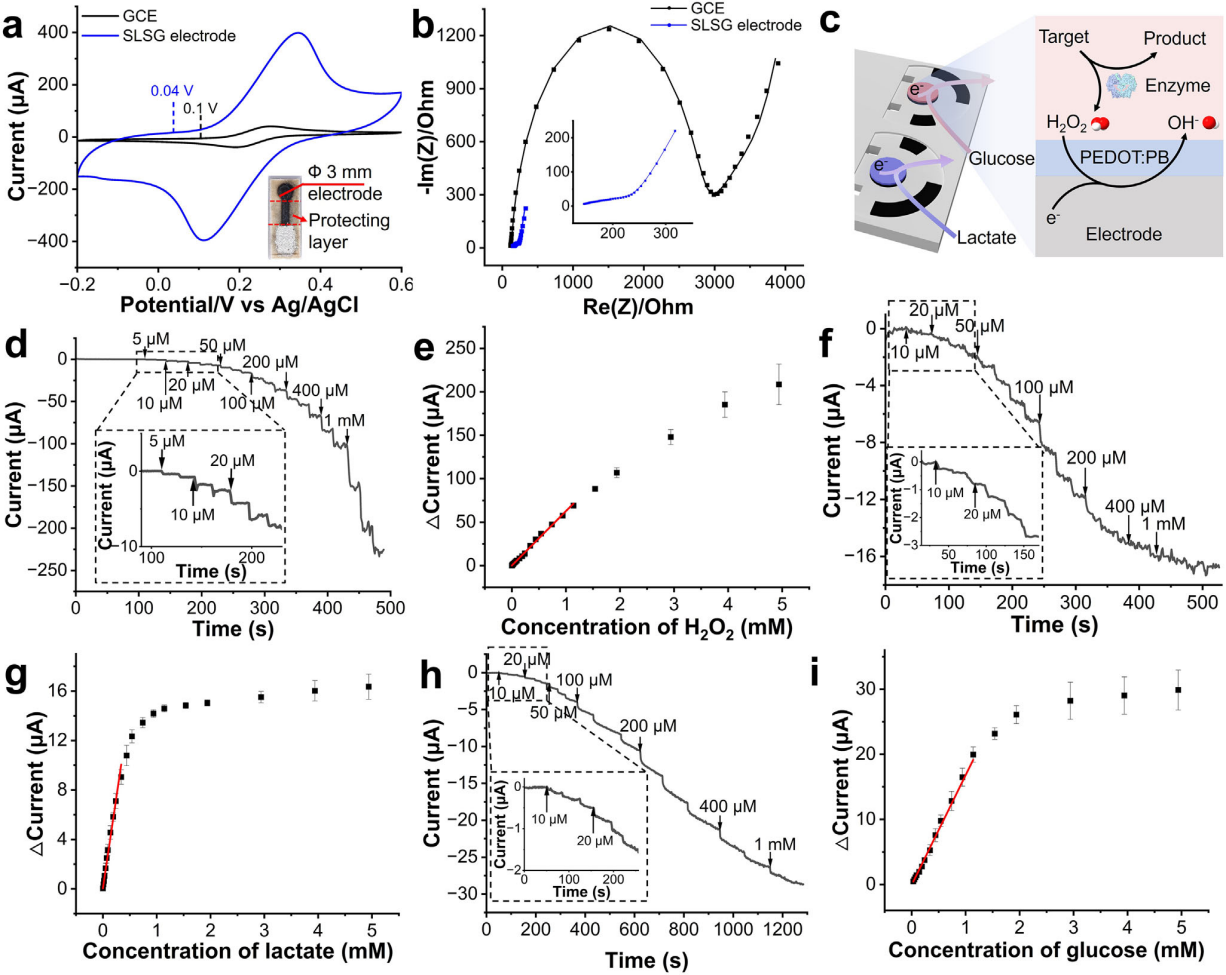
Electrochemical sensing using SLSG electrodes. (a) CVs of the GCE and SLSG electrode in 5 mM Fe(CN)_6_^3−/4−^ in 0.1 M KCl, at a scan rate of 40 mV/s. The inset shows the image of the SLSG electrode. (b) EIS of the GCE and SLSG electrode in 5 mM Fe(CN)_6_^3−/4−^ in 0.1 M KCl over the frequency range 0.1–100 kHz and at 10 mV amplitude. (c) Schematic diagram of the lactate and glucose biosensor. (d) Current−time response curve of PEDOT:PB/SLSG to successive addition of H_2_O_2_ in 0.1 M PBS (pH = 6.4) at 0.03 V and (e) the corresponding calibration curve, *n* = 3. (f) Current−time response curve of LOx/PEDOT:PB/SLSG to successive addition of lactate in 0.1 M PBS (pH = 7) at 0.03 V and (g) the corresponding calibration curve, *n* = 3. (h) Current−time response curve of GOx/PEDOT:PB/SLSG to successive addition of glucose in 0.1 M PBS (pH = 6.4) at 0.03 V and (i) the corresponding calibration curve, *n* = 3.

Figure 3b presents the Nyquist plots from EIS measurements for the SLSG electrode. The SLSG electrode exhibited a semicircle feature in the high‐frequency region related to the charge transfer resistance (*R*_ct_) and a Warburg diffusion line in the low‐frequency region corresponding to the semi‐infinite diffusion‐controlled processes. The equivalent circuit and detailed fitting parameters (e.g. *R*_s_, *R*_CT_, W) are summarized in Figure S11 [72, 73]. The SLSG electrode demonstrates significantly enhanced electrode kinetics, as evidenced by a 20‐fold lower *R*_ct_ value of 123.6 Ω compared to the GCE (2581 Ω), and a 55‐fold lower Warburg coefficient (W) of 51.19 Ω compared to the GCE (2840 Ω). These results indicate a faster electron‐transfer rate and enhanced mass transport [74, 75] for the SLSG electrode due to its high conductivity, large active surface area, and superior electrocatalytic activity, consistent with the CV results [76].

The SLSG electrode was further developed into lactate and glucose biosensors based on functionalization with enzyme/poly(3,4‐ethylenedioxythiophene) (PEDOT) embedded Prussian blue composite (PEDOT:PB) on the SLSG electrode (Figures 3c and S12) [77, 78]. The enzymatic catalysis of lactate or glucose by lactate oxidase (LOx) or glucose oxidase (GOx) generates H_2_O_2_, which is subsequently catalyzed by the PEDOT:PB transducing layer producing current signals. The high catalytic activity of PEDOT:PB for H_2_O_2_ was demonstrated by the current‐time response of the PEDOT:PB/SLSG electrode shown in Figure 3d, measured at 0.03 V with successive additions of H_2_O_2_. The current response increased with increasing H_2_O_2_ concentration, delivering a linear current response as a function of H_2_O_2_ concentration (Figure 3e) over the range of 5–1140 µM (R^2^ = 0.998) with a high sensitivity of 62.54 µA/mM and limit of detection (LOD) of 2.3 µM (3*σ*/sensitivity). The analytical performance for H_2_O_2_ sensing at PEDOT:PB/SLSG was comparable to other PB‐based carbon materials described in the literature (Table S3).

The performance of the LOx/PEDOT:PB/SLSG and GOx/PEDOT:PB/SLSG biosensors in response to lactate and glucose was subsequently investigated. Figure 3f shows the current‐time curve of the LOx/PEDOT:PB/SLSG biosensor upon successive additions of lactate at an applied potential of 0.03 V. The current response (Figure 3g) was linear with the lactate concentration over the range of 0.01–0.34 mM (R^2^ = 0.992) with a sensitivity of 29.43 µA/mM and an LOD of 0.3 µM. Similarly, Figure 3h presents the current‐time curve of the GOx/PEDOT:PB/SLSG biosensor upon successive glucose additions at 0.03 V. The current response (Figure 3i) demonstrated linear behavior over the glucose concentration range of 0.01‐1 mM (R^2^ = 0.994), with a sensitivity of 16.86 µA/mM and an LOD of 3 µM. Notably, the analytical performance of the lactate and glucose biosensors using SLSG electrodes surpassed that of previously reported biosensors employing advanced carbon‐based electrodes, including carbon nanotubes (CNTs), nitrogen‐doped carbon materials, superhydrophobic carbon, and laser‐induced graphene (LIG) (Tables S4 and S5). This excellent performance likely results from the relatively large electroactive surface area of the SLSG electrode, attributed to the interconnected “fluffy” nanostructures that facilitate enzyme efficient immobilization and signal transduction [50].

The robustness of the SLSG‐based biosensor was further evaluated under physiologically relevant conditions. The standard concentrations employed for these tests, 500 µM glucose and 10 mM lactate in 0.1 M PBS, fall within relevant sweat concentration ranges [79, 80]. Reproducibility was assessed using five independently fabricated biosensors in a batch [81]. The SLSG‐based glucose biosensors exhibited inter‐electrode coefficients of variability (inter‐CV) of 6.5% and intra‐electrode CV (intra‐CV) below 1.6% in 500 µM glucose, while the SLSG‐based lactate biosensors exhibited an inter‐CV of 3.3% and intra‐CV below 3.0% in 10 mM lactate (Figure S13), indicating good reproducibility of the SLSG‐based biosensors [82, 83, 84]. Long‐term operational and storage stabilities of the SLSG‐based biosensors were evaluated. Continuous operational stability tests over 2 h [85] showed that the SLSG‐based glucose and lactate biosensors retained 93.7% and 98.1% (7000∼7200 s) of their initial responses (0∼200 s), respectively (Figure S14). Storage stability tests conducted at 4°C under dry and sealed conditions for 0, 1, 3, 5, and 7 days [85] showed the response CVs were 2.6% for glucose and 3.6% for lactate, with minimum retained responses above 92.9% and 95.0%, respectively (Figure S15). These results indicate good long‐term stability of the SLSG‐based biosensors [86, 87]. The anti‐fouling test of 2 h exposure to real human sweat followed by gentle rinsing and retesting in standard analyte solutions [88, 89] showed the SLSG‐based glucose and lactate biosensors retained 90% and 92% of their initial responses, respectively (Figure S16), indicating good anti‐fouling ability of the SLSG‐based biosensors [90, 91]. These results demonstrate that SLSG‐based biosensors provide stable, reproducible, and fouling‐resistant performance compared to representative Pt‐, Au‐, PEDOT:PSS‐, carbon‐based bioelectrodes/biosensors (Table S6).

The integration of sensors/circuits with fluidic components is critical for wearable biosensing devices, as it establishes the sensor‐fluidics interface through biofluid collection, transport, detection, and elution. However, seamless integration between these functionally and geometrically distinct components remains challenging. Conventional fabrication approaches typically require multi‐material or multi‐method, introducing functional challenges arising from post‐integration or material modification (e.g., circuit‐fluidic crosstalk) and inherent geometric complexities of bonding, assembly, and alignment steps.

In this context, we demonstrated a unibody‐designed, watch‐style UMMB (Figure 4) as a wearable biosensing device, fabricated entirely within a single, autonomous SLSG printing process. Rather than an assembled stack of separately fabricated electronic and microfluidic components, the UMMB is a mono‐matrix electro‐fluidic biosensing device in which stereo‐circuits and stereo‐fluidics are embedded and unibody‐integrated as geometry‐defined functional components directly within the monolithic printed geometry. This unibody stereo‐architecture enables 3D routing of both electrical signals and biofluids, with seamless, crosstalk‐free integration within a compact structure. Images of the SLSG‐printed UMMB, schematic diagrams of the UMMB, and its schematic stereo‐circuits and stereo‐fluidics are shown in Figure 4a‐i–iii, respectively. The schematic fabrication process of the UMMB is shown in Figure S17.

**FIGURE 4:**
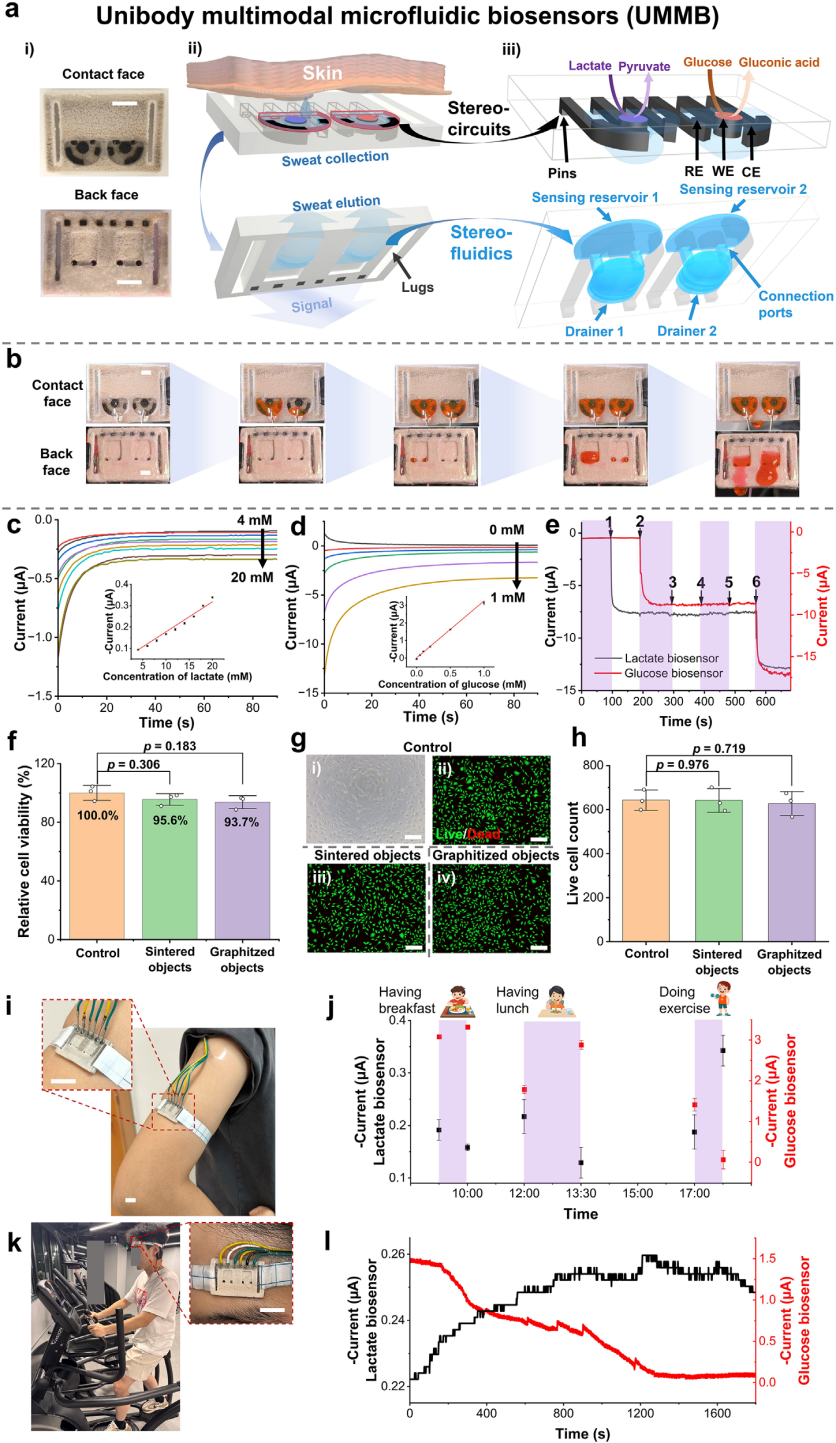
UMMB for metabolites detection and on‐body biosensing. (a) The SLSG printed UMMB (i) and its schematic diagram (ii, iii), scale bar: 5.8 mm. (b) Fluidic test of UMMB, scale bar: 5.8 mm. (c) Amperometric response of the integrated lactate biosensor to the increasing concentration of lactate in the range of 4−20 mM in artificial sweat, and the inset is the calibration curve, *n* = 3. (d) Amperometric response of the integrated glucose biosensor to the increasing concentration of glucose in the range of 0−1 mM in artificial sweat, and the inset is the calibration curve, *n* = 3. (e) Interference study of the UMMB. 1: 0.25 mM lactate, 2: 0.5 mM glucose, 3: 0.05 mM ascorbic acid, 4: 0.1 mM creatinine, 5: 0.1 mM uric acid, 6: 0.5 mM glucose+0.25 mM lactate. (f) Quantitative viability of L929 cells cultured in control, and with sintered objects or graphitized objects after 24 h incubation period. *n* = 3. Statistical analysis was performed by unpaired two‐tailed Student's t test, where *p* < 0.05 represents significant difference. (g) optical (i) and fluorescence images (ii) of L929 cells cultured in control, and fluorescence images of L929 cells cultured with the sintered objects (iii) or graphitized objects (iv) after 24 h incubation period. Green (calcein AM) and red color (propidium iodide) indicate live and dead cells, respectively. Scale bar: 200 µm. (h) Comparison of the viable cell count derived from the live/dead staining assay. *n* = 3. Statistical analysis was performed by unpaired two‐tailed Student's t test, where *p* < 0.05 represents significant difference. (i) Image of the UMMB on a volunteer, scale bar: 1.7 cm. (j) On‐body metabolite monitoring from the volunteer's daily activities by the UMMB. (k) Image of the UMMB worn by a volunteer doing dynamic exercise, scale bar: 1.7 cm. (l) On‐body metabolite monitoring during the volunteer's dynamic exercise by the UMMB.

The wearable UMMB device consists of stereo‐circuits with SLSG lactate and glucose biosensors for metabolite detection in human sweat, stereo‐fluidics for sweat collection and drainage, and lugs for attachment. The stereo‐circuits incorporate two integrated three‐electrode systems with LOx/PEDOT:PB/SLSG or GOx/PEDOT:PB/SLSG as working electrodes (WE), SLSG Ag/AgCl reference electrodes (RE), and SLSG counter electrodes (CE) for multimodal metabolite monitoring, along with pins for signal routing. To facilitate effective collection, transport and elution of sweat, the unibody stereo‐fluidic system is built around the SLSG biosensors and consists of two sensing reservoirs (1 and 2, each with a sweat capacity of ∼30 µL), connection ports, and two drainers (1 and 2) (Figures 4a‐ii, iii and S17g). Figure S18 illustrates the interaction between the stereo‐circuits and stereo‐fluidics, as well as the overall working process of the UMMB. During on‐body monitoring, sweat excreted from the skin gradually fills the sensing reservoirs, glucose and lactate measurements are then initiated while the excess sweat is rerouted through the connection ports to the drainers for drainage and evaporation.

The effectiveness of the stereo‐fluidic system was demonstrated through fluidic tests shown in Figure 4b to simulate the practical sweat collection, transport, and drainage process during wearable use. The red dye solutions were first loaded into the two sensing reservoirs on the contact face of the UMMB, with subsequent filling and transcendence to the external back face directed through the 3D connection ports, followed by drainage and elution by the drainers. The stereo‐fluidic architecture enables directing of excess fluid in the sensing reservoirs through 3D connection pathways to spatially separated drainage zones, promoting sweat elution and respiration during the biosensing operated in stereo‐circuits, which was further clarified by the working‐process schematic in Figure S18 [92]. Additionally, no observable leakage occurred during flow testing, indicating the absence of circuit‐fluidic crosstalk in the UMMB and confirming the effectiveness of the system.

The performance of the UMMB multimodal metabolite biosensing system was investigated using the SLSG lactate and glucose biosensors within the stereo‐circuit in response to lactate and glucose in artificial sweat. The amperometric responses are shown in Figure 4c,d, with calibration curves presented as insets. The analytical dynamic window of the LOx/PEDOT:PB/SLSG working electrode was optimized by adding a diffusion layer consisting of PC/chitosan for detecting physiological lactate concentrations [93]. The amperometric curves exhibit distinct current responses to the dynamic concentration ranges of lactate (4‐20 mM) and glucose (0‐1 mM), which are suitable for human sweat metabolite monitoring (∼4‐20 mM lactate and ∼0.02‐0.6 mM glucose concentration in human sweat) [94]. The current responses were linearly proportional to lactate (R^2^ = 0.991) and glucose concentrations (R^2^ = 0.998) with sensitivities of 0.014 µA/mM for lactate sensing (Figure 4c inset) and 3.230 µA/mM for glucose sensing (Figure 4d inset).

The interference test (Figure 4e) was performed to evaluate the selectivity of the lactate and glucose biosensors against common interferents, including ascorbic acid, creatinine and uric acid in sweat. To replicate physiologically relevant conditions, the test was conducted in artificial sweat with sequential additions of the target analytes and representative interferents at their physiological concentrations, including 0.25 mM lactate, 0.5 mM glucose, 0.05 mM ascorbic acid, 0.1 mM creatinine, 0.1 mM uric acid, and a mixture of 0.5 mM glucose + 0.25 mM lactate [95]. As shown in Figure 4e, the specific lactate and glucose biosensors produced distinct current responses only to their respective target analytes (lactate and glucose), whereas no detectable response was observed upon the addition of the representative interfering species. Moreover, the lactate and glucose biosensors responded independently, with no observable circuit‐fluidic crosstalk, demonstrating the good selectivity and effective multimodal biosensing capability of the UMMB.

In vitro cellular compatibility test was performed using L929 fibroblasts [96] to evaluate the cytotoxicity of the SLSG objects (Figure 4f–h). Figure 4f presents the CCK‐8‐based cytotoxicity assessment of the sintered and graphitized objects (∼6 mm diameters) after 24 h direct co‐culture with L929 fibroblasts. The sintered and graphitized objects maintained high relative cell viabilities of 95.6% and 93.7%, respectively, indicating no significant reduction in relative cell viability compared with the control group (*p* > 0.05). To further validate the CCK‐8 results, optical imaging and live/dead fluorescence staining were conducted after 24 h incubation (Figure 4g). In the live/dead assay, viable cells were indicated by green calcein‐AM staining, whereas dead cells were indicated by red propidium iodide staining. The control, sintered‐object, and graphitized‐object groups all showed predominantly green‐stained cells with no observable red‐stained cells, consistent with the high relative cell viabilities measured by the CCK‐8 assay. Quantification of the live cell numbers (Figure 4h) were performed by counting the green calcein‐AM‐stained cells from the live/dead fluorescence images in Figure 4g‐ii–iv, revealed comparable live cell counts among the control and tested groups further confirming both the sintered and graphitized objects caused negligible cytotoxicity under the 24 h direct‐contact condition. Together, the CCK‐8 assay and live/dead staining results demonstrate good in vitro cellular compatibility of the SLSG objects for wearable applications, consistent with the reported cytocompatibility of PEEK‐based biomaterials [97].

We further demonstrated the on‐body biosensing capability of the UMMB for monitoring metabolite responses from daily activities by having volunteers place it on their arm, wired for signal processing (Figure 4i). The lactate and glucose responses from a volunteer after having breakfast, having lunch, and exercising during the day are illustrated in Figure 4j. Sweat glucose responses were observed to increase following food intake and decrease after exercise [98], while lactate responses remained subtle with food intake and increased dramatically after exercise [99]. On‐body biosensing capability of the UMMB during dynamic exercise was also demonstrated in Figure 4k. The lactate and glucose responses from a volunteer doing dynamic exercise for 30 min on an elliptical machine are illustrated in Figure 4l. Sweat glucose responses were observed to decrease during exercise [98], while lactate responses increased during exercise [99]. This demonstrates the effectiveness of UMMB in on‐body biosensing and monitoring metabolite responses corresponding to daily and dynamic activities, enabled by SLSG's mono‐matrix fabrication with unibody integration of geometry‐defined functional components, including stereo‐circuits and stereo‐fluidics, and circumvents the functional challenges and geometric complexities inherent to conventional multi‐material or multi‐method fabrication. Table S1 benchmarks SLSG against these conventional strategies across materials, stereo‐circuit/stereo‐fluidic capability, fabrication complexity, instrument requirements, manufacturing steps and customization, underscoring the functional‐geometric fabrication advantages of the SLSG approach.

## 3 Conclusions

In summary, we have successfully developed a novel SLSG 3D printing technology that enables the seamless integration of stereo‐circuits and stereo‐fluidics in wearable integrated electronic devices. This technology achieves unibody designs with tailored wearable features through mono‐matrix autonomous fabrication. SLSG employs adaptive photonic modulation of a carbon‐rich precursor (PEEK) to simultaneously create insulating sintered structures and conductive graphitized patterns through controlled and optimized laser parameters. Low laser power (2.9 W) induces particle sintering for structural components, while high laser power (5.7 W) drives photothermal graphitization, achieving superior graphitization quality, as verified by FTIR, XPS, XRD, and Raman analyses, and conductivity of up to 1895 S/m, surpassing that of conventional laser‐induced graphitization. This superior conductivity contributes to a 7.64‐fold enhancement in the electrochemical performance of SLSG electrodes compared to classic GCE, as confirmed by CV measurements, and is attributed to the interconnected “fluffy” nanostructures and multilayer graphite flake architecture revealed by SEM. We demonstrated the technology's application through a compact, watch‐type UMMB device featuring integrated stereo‐architecture with 3D routing of both electrical signals and biofluids for sweat metabolite monitoring, manipulation and respiration (collection, transport, and drainage). The UMMB achieves sensitivities of 29.43 µA/mM and 16.86 µA/mM with LODs of 0.3 µM and 3 µM for lactate and glucose detection, respectively, exhibiting good sensor robustness, no detectable response to common interfering electroactive species in sweat and no observable circuit‐fluidic crosstalk, thereby demonstrating effective biosensing performance for practical applications. The UMMB highlights the SLSG technology's transformative unibody approach in resolving critical fabrication challenges for microfluidic‐integrated wearable electronics. This demonstration showcases the technology's broad applicability to compact biomedical devices, wearable sensors/biosensors, actuators, and consumer electronics, with promising customizability and personalization capabilities.

## 4 Experimental section

### 4.1 Materials

The PEEK powder and the thermoplastic polyurethane (TPU) powder were purchased from BASF, Germany; both powders have a particle size of ∼25 µm. The polycarbonate (PC) membrane (0.2 µm) used as an encapsulation layer for the lactate biosensor was purchased from Whatman nuclepore, China. 3,4‐ethylenedioxythiophene (EDOT), iron(II) chloride (FeCl_2_), bovine serum albumin (BSA), hydrogen peroxide (H_2_O_2_), glucose, lactate, potassium ferricyanide (K_3_[Fe(CN)_6_]), potassium ferrocyanide (K_4_[Fe(CN)_6_]), potassium hydrogen phosphate (K_2_HPO_4_∙H_2_O), potassium dihydrogen phosphate (KH_2_PO_4_), and others were all purchased from Sigma‐Aldrich, USA and employed without further purification. Phosphate buffer solution (PBS, pH = 6.4 and 7) was prepared by combining KH_2_PO_4_ and K_2_HPO_4_. Silver/silver chloride (Ag/Ag/Cl) ink were purchased from Canrd, China, and Tecos Technology, China, respectively. Deionized (DI) water was obtained using the Milli‐Q System from Merck, USA. Lactate oxidase (LOx, 93.6 U/mg) was obtained from Toyobo, Japan; glucose oxidase (GOx, 24.89 U/mg) was purchased from Sigma‐Aldrich, USA.

### 4.2 SLSG Process and Optimalization of SLSG Printing Parameters

SLSG printing was performed at room temperature (∼25°C) using an SLSG fabrication platform (details in Supporting Information) with PEEK powder (100%) and/or PEEK+TPU (70%:30%) composite mixed by a mechanical agitator for 1 h followed by manual stirring for 10 min to ensure proper mixing. Glass substrates (35×35 mm) with 1 mm‐thick TPU tape served as the printing base for SLSG objects. Support structures were incorporated when printing overhanging structures to prevent collapse.

The SLSG system employed a 10.6 µm CO_2_ Gweike Cloud RF metal tube laser cutter purchased from Gweike Cloud, China, with a maximum laser power of 38 W and a settled PPI of 508 controlled by Lightburn software. Laser parameters including scan speed (from 25 to 200 mm/s), laser power (from 2.00 to 6.84 W), and laser defocus distance (from 0 to 10 mm) were studied. The final laser parameters for SLSG printing were optimized at 100 mm/s laser scan speed for sintering and graphitization, 2.90 W and 0 mm defocus distance for sintering, and 5.70 W and 6 mm defocus distance for graphitization. The layer feeding thickness controlled by the custom‐built 3D printing platform ranged from 0.1 to 0.3 mm. The final printing feeding layer thickness is 0.1 mm.

### 4.3 Fabrication of SLSG Stereo‐circuit and Stereo‐fluidic

The SLSG stereo‐fluidic and stereo‐circuit were designed using CAD, followed by conversion to G‐code and uploading to the SLSG fabrication platform. The printing process was then initiated, where the automated feeding system coated the powder bed, and the CO_2_ laser head wrote following the corresponding set laser parameters and designed patterns with 0.1 mm feeding layer thickness to conduct sintering and graphitization (details in Supporting Information). After printing, the stereo‐circuit and stereo‐fluidic objects were removed from the glass‐TPU substrate, followed by ultrasonic treatment in an ethanol bath for 1 min to remove remaining powder, and then dried in an oven at 60°C for 20 min.

For the SLSG stereo‐circuits, the graphitized electrodes were treated before use with electrochemical activation by chronoamperometry in 0.5 M NaOH at +1.4 V for 200 s and then at ‐1.0 V for 200 s, followed by activation by cyclic voltammetry (CV) in 0.1 M HCl and 0.1 M KCl from ‐0.2 to 0.5 V at 40 mV/s for 10 cycles using a PalmSens 4 potentiostat, Netherlands, with an external platinum (Pt) wire/plate as CE and Ag/AgCl (3 M KCl) as external RE.

### 4.4 Characterizations of the SLSG Printed Stereo‐Circuit and Stereo‐Fluidic

The pattern, structure, morphology, and material properties of the printed SLSG objects were characterized by optical microscopy, SEM, energy‐dispersive x‐ray spectroscopy (EDS), and Raman spectroscopy. Optical images of the printed SLSG objects were taken using an optical microscope (ZEISS Axiolab 5, Germany). Structure and morphology were characterized by SEM (ZEISS Sigma 360 at 2.0 kV, Germany). The SEM was equipped with an EDS analyzer to determine the elemental composition of SLSG objects. Cross‐section SEM images of the SLSG objects were captured after either immersing the objects in liquid nitrogen for 1 min followed by breaking them in the center by force, or directly breaking the samples in the center by force without liquid nitrogen pretreatment. Material properties of the SLSG objects were characterized by FTIR spectroscopy, XPS, XRD, and Raman spectroscopy. FTIR spectroscopy was performed using a Nicolet iN10 (Thermo Fisher Scientific, USA) equipped with an attenuated total reflection (ATR) measuring cell. XPS was acquired with a K‐Alpha (Thermo Fisher Scientific, USA) equipped with a monochromatized Al Kα light source (1486.6 eV). Quantification and deconvolution of the high‐resolution C 1s spectra were performed with the Avantage software (Thermo Fisher Scientific, USA) and specialized algorithms. XRD patterns were obtained using a D8 Venture (Bruker, Germany) with Cu Kα radiation over 10–60° using 2°/min. Raman spectroscopy was performed with a confocal Raman microscope (CRM) (Alpha300R, WITec GmbH, Germany) equipped with a TEM single‐frequency laser (*λ* = 532 nm, laser power = 40 mW, WITec GmbH, Germany). The graphitized samples used for FTIR, XPS, and XRD characterizations were in the powder form scraped from the surface of SLSG graphitized objects.

Electrochemical characterizations of SLSG electrodes were conducted using a PalmSens 4 potentiostat, Netherlands. CV and EIS of SLSG working electrode (WE, 3 mm diameter, details in Figure S17g), glassy carbon electrode (GCE, 3 mm diameter), and screen‐printed carbon electrode (SPCE, 4 mm diameter, Metrohm, DRP‐110, Spain) were conducted in 0.1 M KCl containing 5 mM Fe(CN)_6_^3−/4−^ with external Ag/AgCl RE and Pt CE.

The resistance of graphitized objects was measured using an ohmmeter purchased from DELIX, China. The in‐plane conductivity of graphitized objects was measured using a four‐probe conductivity meter purchased from REOTAI, China. Details of resistance, in‐plane conductivity, thickness, and through‐plane conductivity measurements are provided in the SI. The thickness of sintered and graphitized products was measured using a thickness gauge purchased from Syatek, China, with a precision of 0.01 mm. Flow tests of the stereo‐fluidics were performed using a syringe pump (Thermo Fisher Scientific, USA) with colored dye as fluid at a flow rate of 0.5 mL/min; TPU transparent tapes were used for sealing.

Cytotoxicity assessment and the live/dead staining experiment were conducted using L929 cells (CL‐0137, Pricella Biotechnology, China). The sintered and graphitized objects were sterilized by autoclave sterilization at 121°C for 20 min. For the cytotoxicity assessment, cells were seeded within a 24‐well plate at a density of 4×10^4^ cells, 1000 µL/well and incubated at 37°C in a humidified atmosphere with 5% CO_2_. Samples (∼6 mm diameters) were added to wells of the tested groups to directly contact with cells. After the 24 h incubation period, the cells in each well were washed three times by PBS and were then added with 1000 µL 10% CCK‐8 solution (IV08‐100, Invigentech, China) and further incubated for 2 h at 37°C with 5% CO_2_. The absorbance at 450 nm was then measured using a microplate reader (SPARK 10 M, TECAN, Switzerland) for evaluation. For the live/dead staining experiment, cells were seeded within confocal dishes at a density of 1×10^5^ cells, 1000 µL/dish, and incubated at 37°C in a humidified atmosphere with 5% CO_2_. Samples (∼6 mm diameters) were added to dishes of the tested groups to directly contact with cells. After the 24 h incubation period, the cells were treated with Calcein‐AM/propidium iodide (BB‐4126, Bestbio, China) for 15 min to stain the live and dead cells. Optical and fluorescence images of the cells were recorded using an optical/fluorescence microscope (Axio Vert.A1, ZEISS, Germany).

### 4.5 Fabrication of Integrated UMMB for on‐body Sensing

The integrated UMMB was designed using CAD with a unibody design approach to seamlessly integrate stereo‐circuits and stereo‐fluidic. The UMMB was designed as a watch‐type device with lugs and flexible watch band for effective mounting onto skin for wearable sensing. The stereo‐circuits consist of two three‐electrode systems (detailed design layout shown in Figure S17) with bare SLSG electrode as CE and Ag/AgCl ink‐modified SLSG electrode as RE (Ag/AgCl ink stencil‐printed on the RE surface followed by curing on a hotplate at 120°C for 2 min). The WE of the printed UMMB was functionalized with PEDOT:PB via one‐pot electrochemical deposition [100] by CV scanning from −0.5 to 1.2 V in 0.1 M HCl containing 10 mM EDOT monomer and a 10 mM mixture of K_4_[Fe(CN)_6_] and FeCl_2_ for 20 cycles at a scan rate of 50 mV/s to obtain the PEDOT:PB/SLSG electrode.

The UMMB consists of multimodal glucose and lactate biosensor for metabolite monitoring. For the preparation of the glucose‐sensing UMMB, an aliquot of glucose oxidase solution (3 µL) containing 20 mg/mL glucose oxidase (497.8 U/mL) with 10 mg/mL BSA was deposited onto one of the PEDOT:PB/SLSG electrodes and dried at 4°C for 2 h, followed by the addition of 2 µL of chitosan solution (1 wt% in 1% acetic acid) to obtain the enzyme‐functionalized GOx/PEDOT:PB/SLSG electrode. For the preparation of the lactate‐sensing UMMB, the other PEDOT:PB/SLSG electrode was functionalized with lactate oxidase (3 µL containing 40 mg/mL lactate oxidase (3744 U/mL) with 10 mg/mL BSA) following the same procedure to obtain the LOx/PEDOT:PB/SLSG electrode. The LOx/PEDOT:PB/SLSG electrode was then incorporated with a PC membrane wetted with chitosan solution (1% in 1% acetic acid) as the diffusion layer to obtain the functionalized UMMB. The functionalized UMMB was stored at 4°C in a sealed Petri dish when not in use and was hydrated with either PBS (pH 7.0 for lactate, pH 6.4 for glucose) or artificial sweat on the electrode area before measurement.

### 4.6 Analytical Performance of UMMB

The analytical performance of the PEDOT:PB/SLSG, LOx/PEDOT:PB/SLSG, and GOx/PEDOT:PB/SLSG electrodes for detecting H_2_O_2_, lactate, and glucose was evaluated by amperometry under stirring conditions with external RE and CE in 0.1 M PBS (pH = 7 for lactate, pH = 6.4 for glucose and H_2_O_2_) loaded with different concentrations of H_2_O_2_, lactate, and glucose. The robustness tests of the LOx/PEDOT:PB/SLSG, and GOx/PEDOT:PB/SLSG biosensors for detecting 10 mM lactate and 500 µM glucose were evaluated by chronoamperometry with external RE and CE in 0.1 M PBS (pH = 7 for lactate, pH = 6.4 for glucose). The real human sweat used in the anti‐fouling test was obtained from three volunteers in a batch. The analytical performance of the integrated UMMB was conducted by chronoamperometry in artificial sweat (containing 0.5% NaCl, 0.1% KCl, 0.1% urea with pH = 6.5, following European standard EN1811:2012) loaded with different concentrations of lactate and glucose.

The on‐body test of the UMMB was performed using three human subjects in compliance with the ethical regulations under protocols (CREC: 2025.276) approved by the Joint CUHK‐NTEC Clinical Research Ethics Committee. All subjects gave written informed consent before participating in the study. For the full‐day evaluation, subjects were tasked with performing various activities over a 9‐hour period. For the dynamic‐exercise evaluation, the UMMB was worn by one human subject during 30 min of elliptical‐machine exercise. During on‐body tests, subjects cleaned their skin with water and alcohol swabs before attaching UMMB. The UMMB was connected to and controlled by a portable sensing system and secured to ensure stable on‐body operation, with testing beginning after sweat emerged from the drainers. No discomfort or adverse skin reactions were reported during testing.

## Author Contributions

**Li Zhang**: methodology, writing – original draft, investigation, validation, formal analysis. **Wing Cheung Mak**: conceptualization, writing – review and editing, investigation, methodology, supervision, formal analysis.

## Conflicts of Interest

The authors declare no conflict of interest.

## Supporting information




**Supporting File**: adma75075‐sup‐0001‐SuppMat.pdf.

## Data Availability

The data that support the findings of this study are available from the corresponding author, Wing Cheung Mak, upon reasonable request.
