# Supplementary material for: Selective Laser Sintering and Graphitization for Mono‐Matrix Unibody Fabrication of Integrated Stereo‐Circuit and Stereo‐Fluidic Wearable Electronics
Source: Adv Mater. 2026 Sep 21;38(62):e75075. doi: 10.1002/adma.75075 (PMC13741099; doi:10.1002/adma.75075)
Supplement: Supplementary file 1 [file ADMA-38-e75075-s001.pdf]

Supporting Information

for

Selective Laser Sintering and Graphitization for Mono-Matrix Unibody Fabrication of Integrated Stereo-Circuit and Stereo-Fluidic Wearable Electronics

Li Zhang[†], **Wing Cheung Mak**[†*]

[†]*Department of Biomedical Engineering, The Chinese University of Hong Kong, Hong Kong, China*

[*]*Corresponding Author, e-mail: wing.cheung.mak@cuhk.edu.hk*

Note 1. In-plane conductivity and through-plane conductivity measurement

In-plane conductivity: The average sheet resistance of SLSG graphitized objects (24 mm × 24 mm, single layer, geometrical correction coefficient: 0.96) was measured three times using a four-probe conductivity meter purchased from REOTAI, China. The conductive layers of the SLSG objects were scraped off to measure their thickness using a thickness gauge purchased from Syatek, China with a precision of 0.001 mm. The in-plane conductivities of the SLSG objects were then calculated from the thickness of the conductive layers and corresponding average sheet resistance using the 4-probe technique.[1]

Through-plane conductivity: The electrical resistance of SLSG graphitized objects (width × length × height = 7.5 × 7.5 × 2.5 mm, 25 layers) was measured three times using an ohm gauge purchased from DELIX, China. To ensure stable testing and contact, a pair of clamps with conductive foam were attached to both ends. Contact resistance was normalized before measurement. The through-plane conductivities of the SLSG objects were then calculated from the electrical resistance, cross-sectional area, and height of the objects.[2]

Note 2. SLSG fabrication platform and SLSG process

The SLSG fabrication platform was manufactured from aluminum alloy using computer numerical control (CNC), while the custom-built automated feeding system was fabricated using a Formlabs Form 4 SLA 3D printer with high-temperature resin from Formlabs, China. The automated feeding system comprises a printing chamber, a vibration-assisted blade, and counter-rotating rollers. The printing chamber has a printing area of 35 mm × 35 mm and a height of 27 mm, controlled by stepper motors with drivers purchased from ZJHAY, China with a minimum step size of 0.1 mm. The powder is stored in the vibration-assisted blade, which contains a chamber that vibrates during recoating to dispense powder onto the powder bed. Two counter-rotating rollers are driven by a stepper motor for powder recoating.

During the SLSG process, the stereo-circuits and stereo-fluidics employ a unibody design approach using computer-aided design (CAD), which is subsequently converted to G-code and uploaded to the SLSG printing platform. The autonomous SLSG printing process commences with the vibration-assisted blade and counter-rotating rollers moving across the top of the printing chamber while depositing a flat powder bed, then returning to the starting position. The CO_2 laser head then writes according to preset laser parameters and designed patterns to achieve selective sintering and graphitization. Upon completion of each layer, the printing chamber descends by a specified layer thickness before the blade recoats the subsequent layer. This process is repeated until the final SLSG-printed objects are obtained.

Note 3. Laser defocus distance and laser spot size

The focusing distance determines the laser spot size and is specific to each laser system. For our CO_2 Gweike Cloud RF metal tube laser cutter purchased from Gweike Cloud, China, the laser spot size at the focus state (0 mm defocus distance) is 0.07 mm in diameter. Since the CO_2 laser has a Gaussian intensity distribution,[3] adjustment of the laser spot size can be achieved directly by altering the defocus setting. Here, we used the relative displacement of the laser lens from its position at the focus state as the defocus distance. The focus lens has a diameter of 15 mm and a focal length of 76.2 mm, while the laser beam generated by the machine has a diameter of 8 mm. From the focus state to 10 mm defocus, the laser spot size increases. The larger the laser spot size, the larger the laser scanning area, resulting in greater overlap between laser lines.[4]

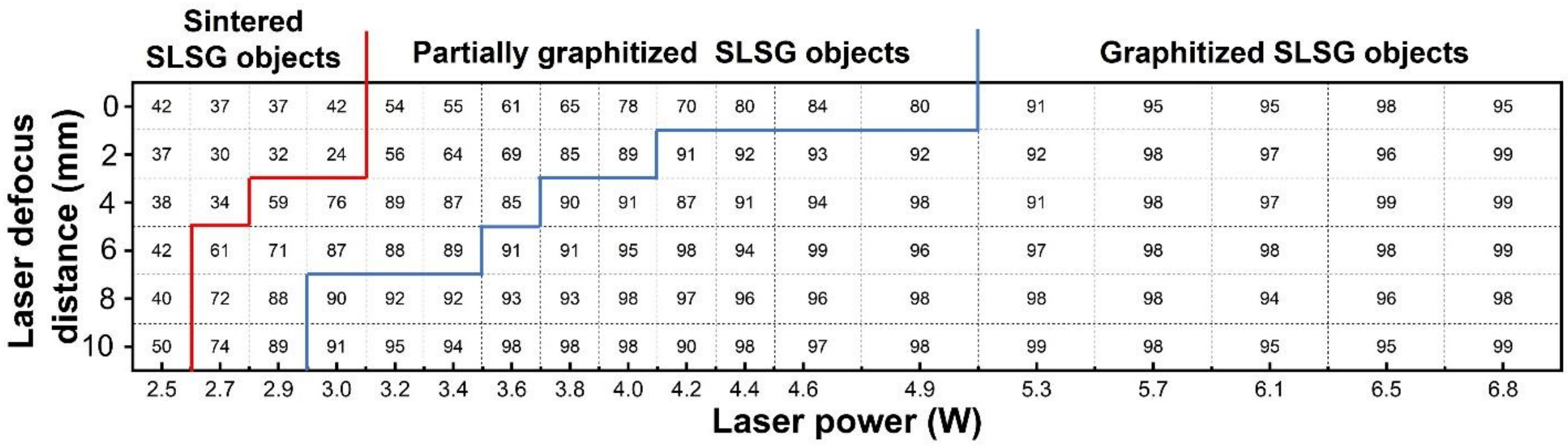

Figure S1. Corresponding gray scale values of the SLSG objects in Figure 1a(i).

a

Sintered and partially graphitized SLSG objects | Graphitized SLSG objects

Laser defocus distance (mm) \ Laser power (W)	2.5	2.7	2.9	3.0	3.2	3.4	3.6	3.8	4.0	4.2	4.4	4.6	4.9	5.3	5.7	6.1	6.5	6.8
0	N.A.	N.A.	N.A.	N.A.	N.A.	N.A.	N.A.	N.A.	N.A.	N.A.	37333	89667	3302	66	34	21	24	23
2	N.A.	N.A.	N.A.	N.A.	N.A.	N.A.	N.A.	N.A.	N.A.	334	427	115	53	22	19	18	37	14
4	N.A.	N.A.	N.A.	N.A.	N.A.	N.A.	2612	172	101	50	36	57	22	18	17	14	16	14
6	N.A.	N.A.	N.A.	N.A.	N.A.	N.A.	94	53	33	23	26	25	22	18	14	14	12	13
8	N.A.	N.A.	N.A.	303	129	91	48	37	29	24	26	27	22	18	20	13	13	14
10	N.A.	N.A.	N.A.	527	101	91	57	44	54	28	29	23	35	18	18	15	12	18

b

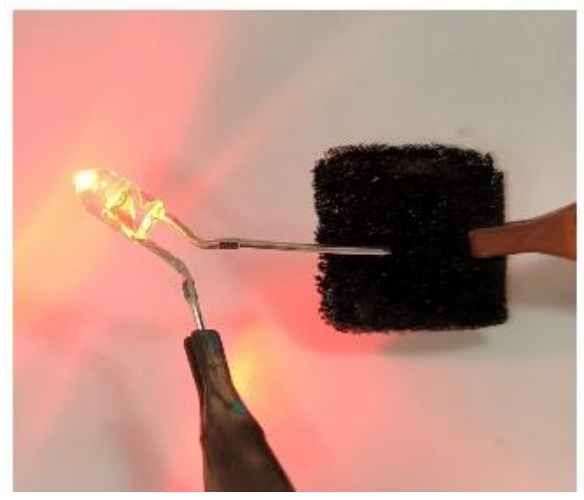

Figure S2. (a) Corresponding electrical resistances of the SLSG objects in Figure 1a(i). the "N.A" means the resistance is infinite. (b) LED testing of a graphitized object.

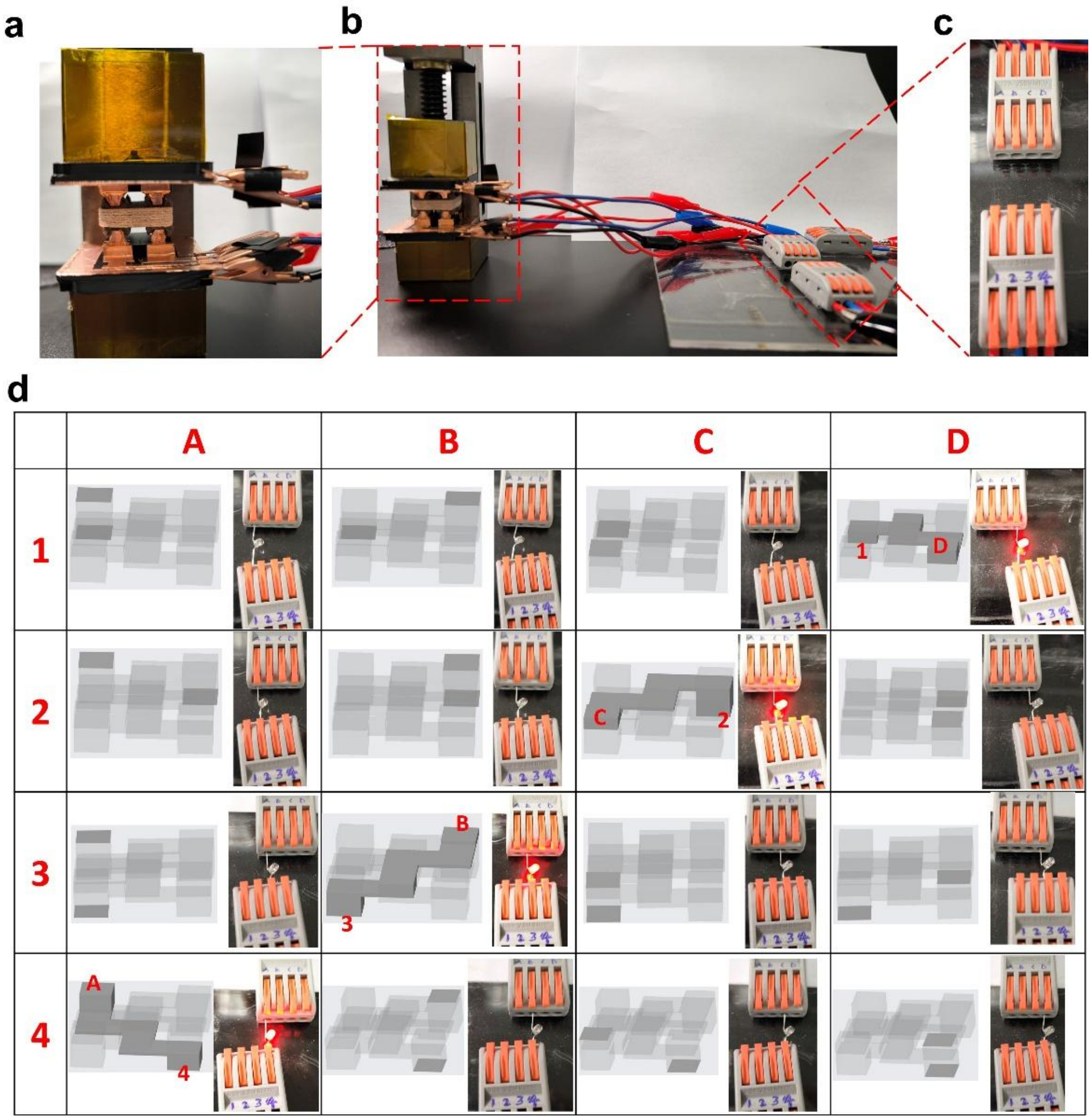

Figure S3. (a) Test bench for testing the SLSG-printed stereo-circuit hub. The test bench has a 3D printed clamp with 4 contacting pins on both the upper and lower part. The four pins on the upper part connect electrode A, B, C and D respectively, while the four pins on the lower part connect electrode No. 1, 2, 3 and 4 respectively. (b) Test bench testing the stereo-circuit hub. (c) Two terminals with eight channels labeling A, B, C, D and No. 1, 2, 3, 4 connected to the eight pins of the test bench. (d) Connection testing of the stereo-circuit hub. 16 connections are shown in the table, each connection was applied 5 V to drive a red LED, only the complementary connections can light up the LED.

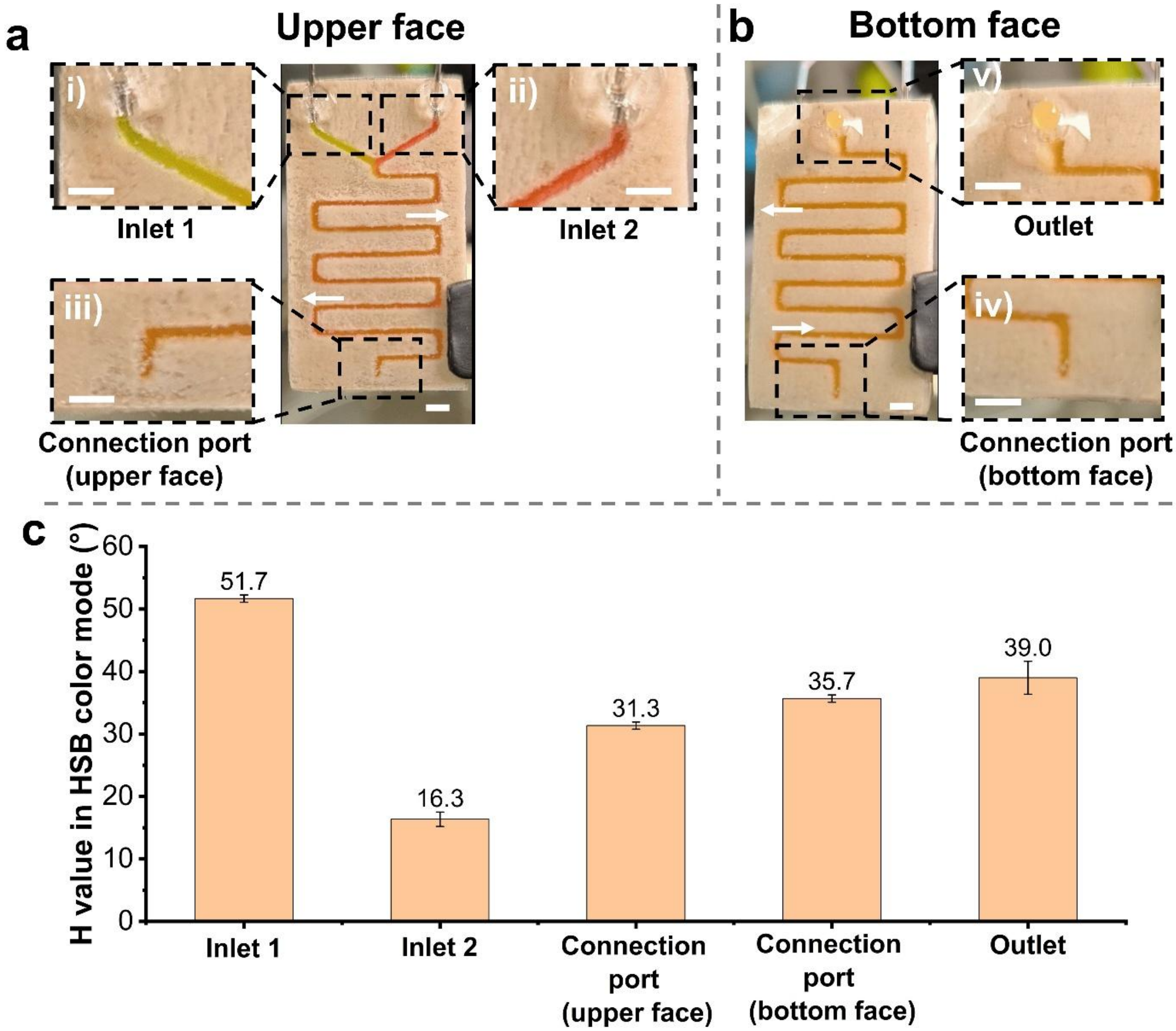

Figure S4. Detailed fluidic mixing and color analysis of the SLSG-printed stereo-fluidic device. (a) Optical images of the upper face during the yellow/red dye mixing test, with enlarged views of inlet 1 (i), inlet 2 (ii), and the upper-face connection port (iii). (b) Optical images of the bottom face after fluid transfer through the connection port, with enlarged views of the bottom-face connection port (iv) and outlet (v). White arrows indicate the flow direction. Scale bar: 2.9 mm. (c) H values in the HSB color model extracted from the optical images at representative fluidic positions, including inlet 1, inlet 2, the upper-face connection port, the bottom-face connection port, and the outlet, showing the color evolution along the stereo-fluidic channel.

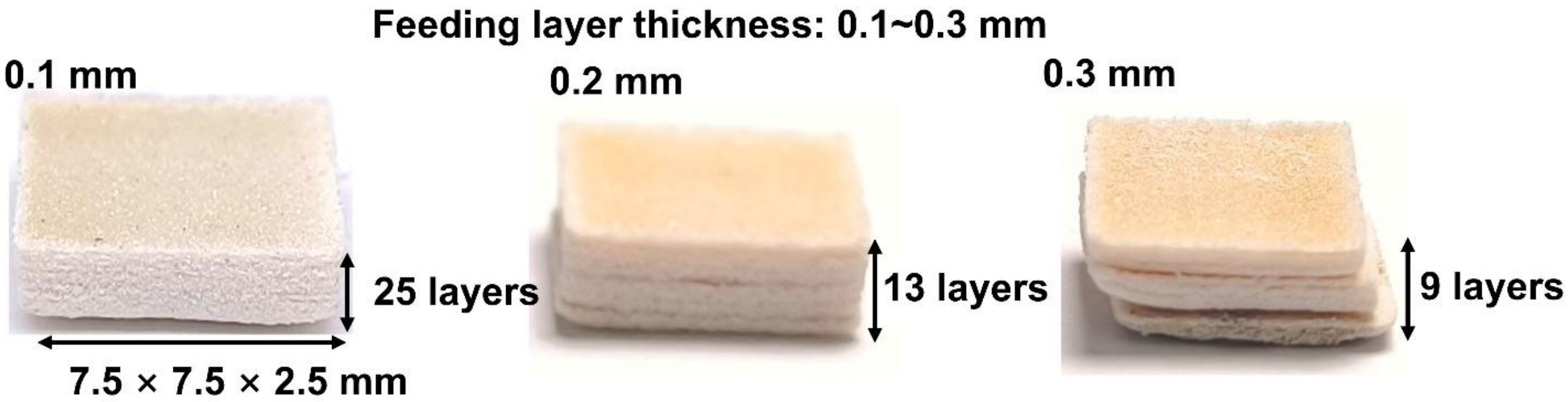

Figure S5. Optical images of sintered objects fabricated with feeding layer thicknesses of 0.1, 0.2, and 0.3 mm under optimal laser parameters. The corresponding layer numbers were 25, 13, and 9, respectively. The designed object dimension is 7.5 × 7.5 × 2.5 mm.

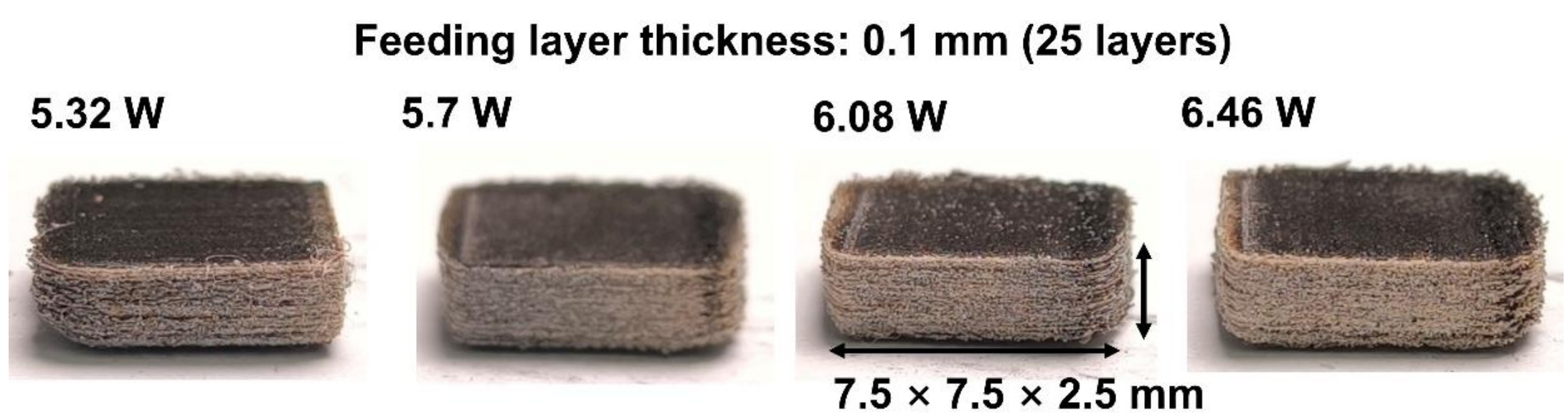

Figure S6. Optical images of graphitized objects fabricated with the 0.1 mm feeding layer thickness (25 layers) under different laser powers. The designed object dimension is 7.5 × 7.5 × 2.5 mm.

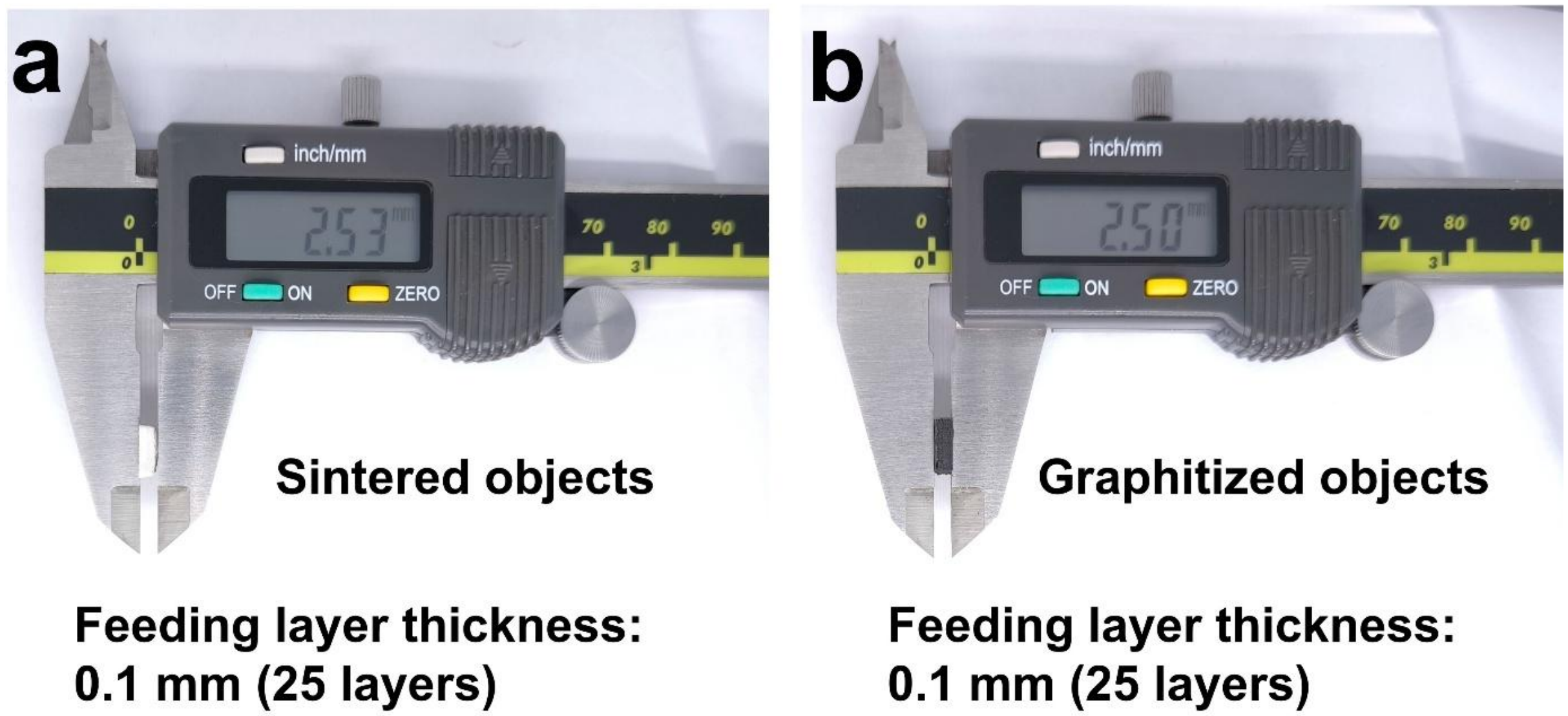

Figure S7. Thickness measurements of multi-layer SLSG objects by a thickness gauge (precision 0.01 mm).

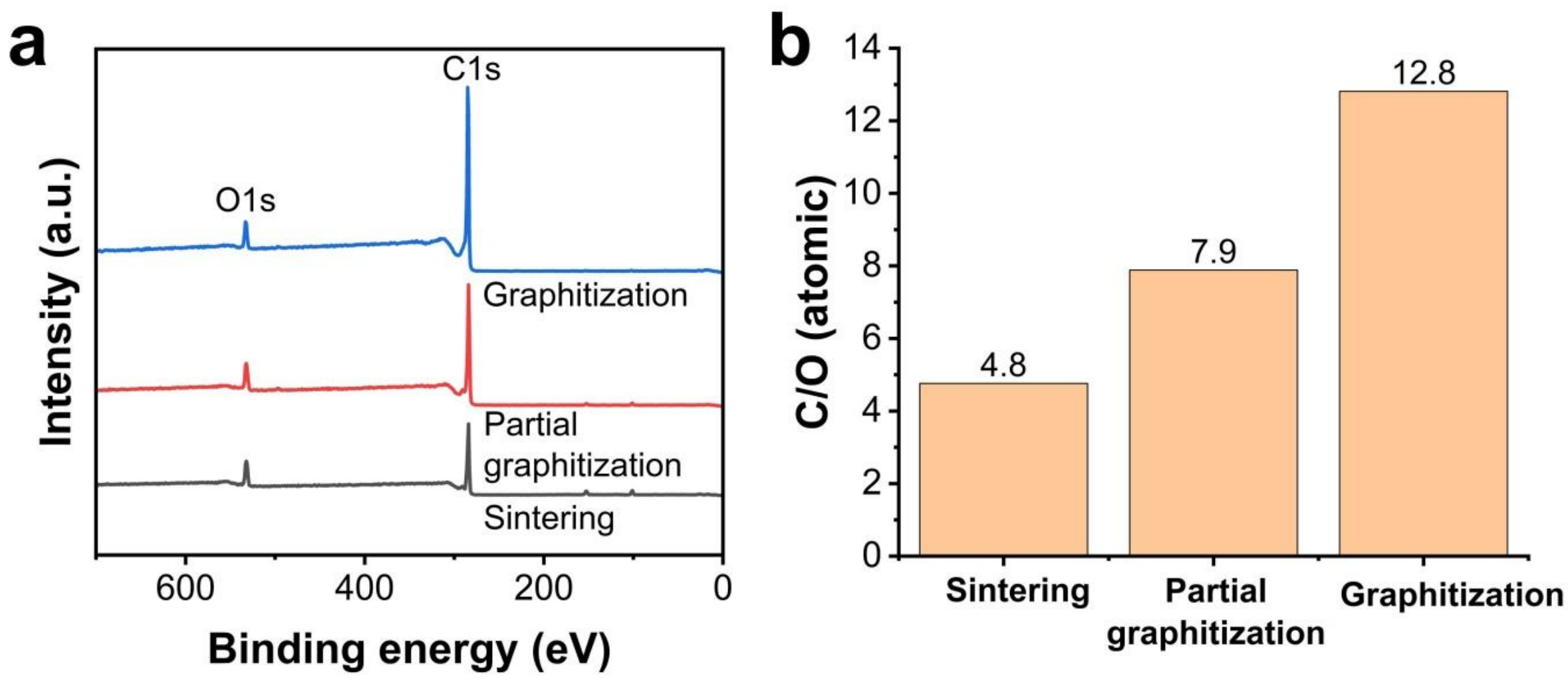

Figure S8. (a) XPS survey spectra of sintered, partially graphitized and graphitized samples. (b) C/O ratio of different samples.

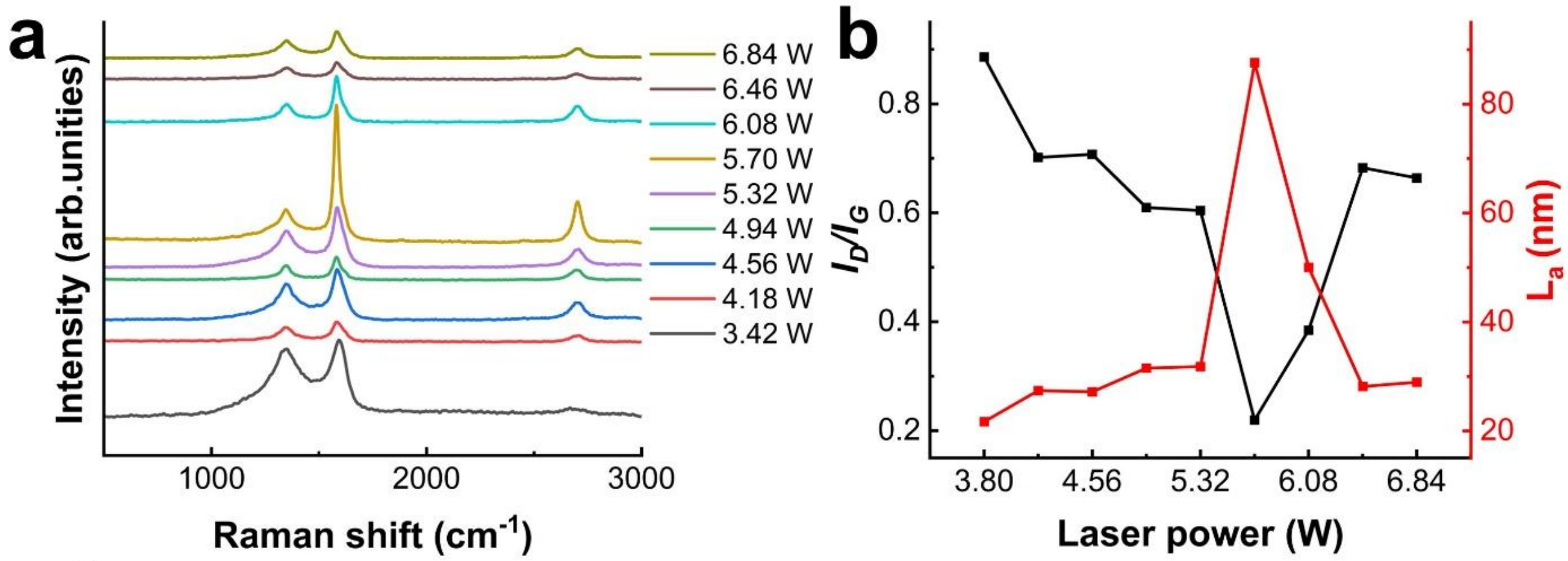

c

Laser power (W)	I_D/I_G	L_a	I_{2D}/I_G
**3.42 W**	0.89	21.69	0.08
**4.18 W**	0.70	27.40	0.31
**4.56 W**	0.71	27.18	0.31
**4.94 W**	0.61	31.53	0.42
**5.32 W**	0.60	31.83	0.27
**5.70 W**	0.22	87.59	0.30
**6.08 W**	0.38	50.03	0.34
**6.46 W**	0.68	28.16	0.30
**6.84 W**	0.66	28.95	0.33

Figure S9. (a) Raman spectra of SLSG objects fabricated under 6 mm defocus and different laser power. (b) Corresponding I_D/I_G and L_a curve of SLSG objects with different laser power. (c) Corresponding I_D/I_G, L_a and I_{2D}/I_G of SLSG objects with different laser power.

Average in-plane crystallite/grain size of graphite/graphene (L_a) is calculated from the I_D/I_G with Equation (1):[5]

$$L_a = 2.4 \times 10^{-10} \times \lambda_{excitation\ laser} \times \left(\frac{I_D}{I_G}\right)^{-1} \quad (1)$$

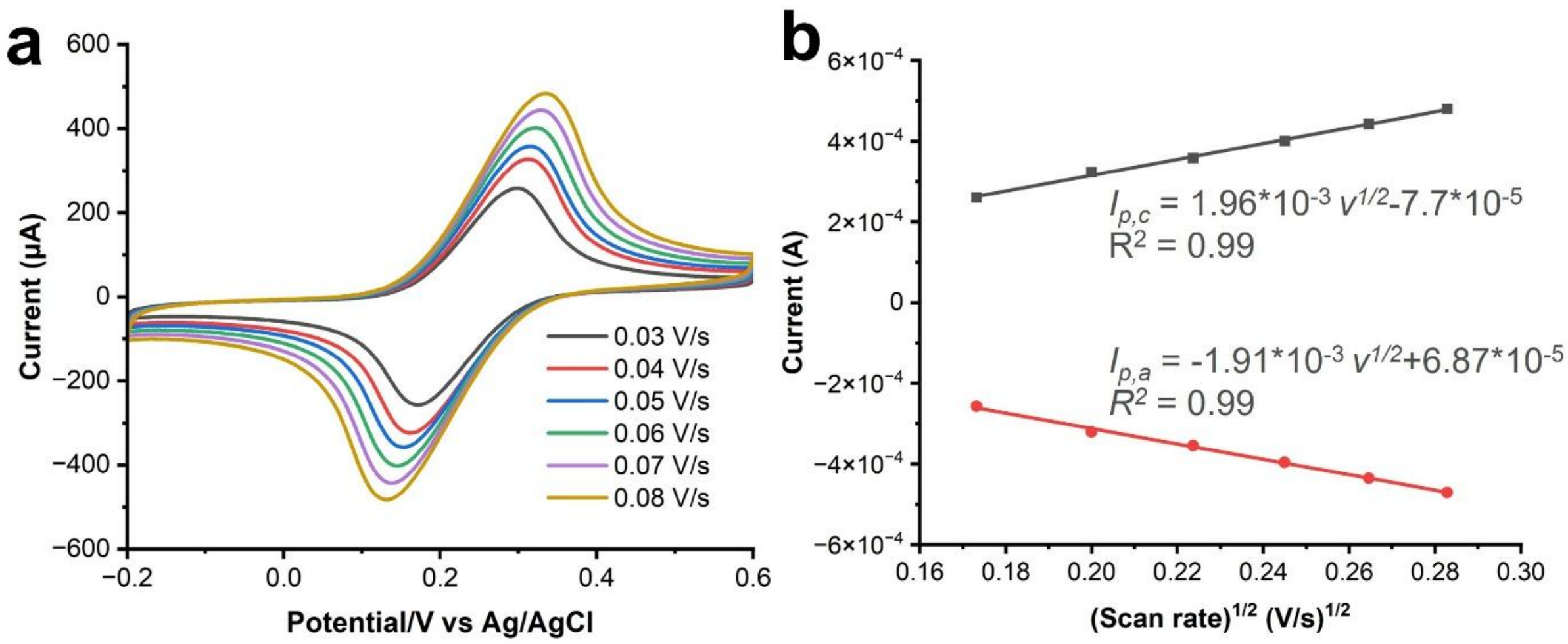

Figure S10. (a) CVs of SLSG electrode in 5 mM $Fe(CN)_6^{3-/4-}$ in 0.1 M KCl with different scan rates from 0.03 to 0.08 V/s. (b) Plots of the $I_{p,a}$ and $I_{p,c}$ versus the square root of scan rates.

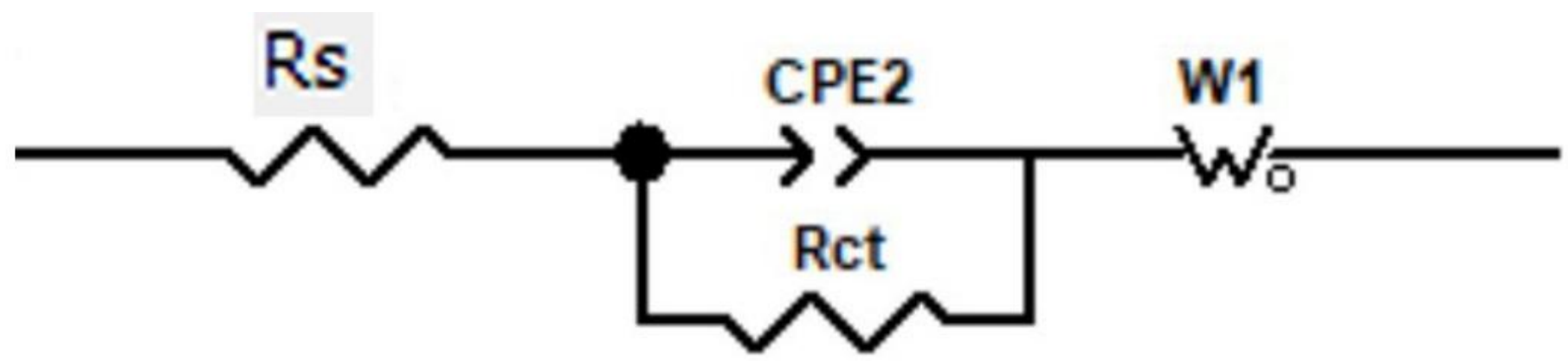

Electrode	R_s (Ω)	R_{CT} (Ω)	Q (μs$^{\alpha}$/Ω)	W (Ω)
GCE	110.1	**2581**	0.5558	2840
SLSG electrode	133.4	**123.6**	2283.4	51.19

Figure S11. Equivalent circuit fitting model and corresponding EIS fitted parameters of GCE and SLSG electrode in 5 mM $Fe(CN)_6^{3-/4-}$ in 0.1 M KCl.

The EIS data (Figure 3b) for GCE and SLSG electrode in 5 mM $Fe(CN)_6^{3-/4-}$ in 0.1 M KCl was fitted by Randles circuit, including a solution resistance (R_s), a charge-transfer resistance (R_{ct}), a Warburg coefficient (W) and a constant phase element (CPE, Q).[6,7]

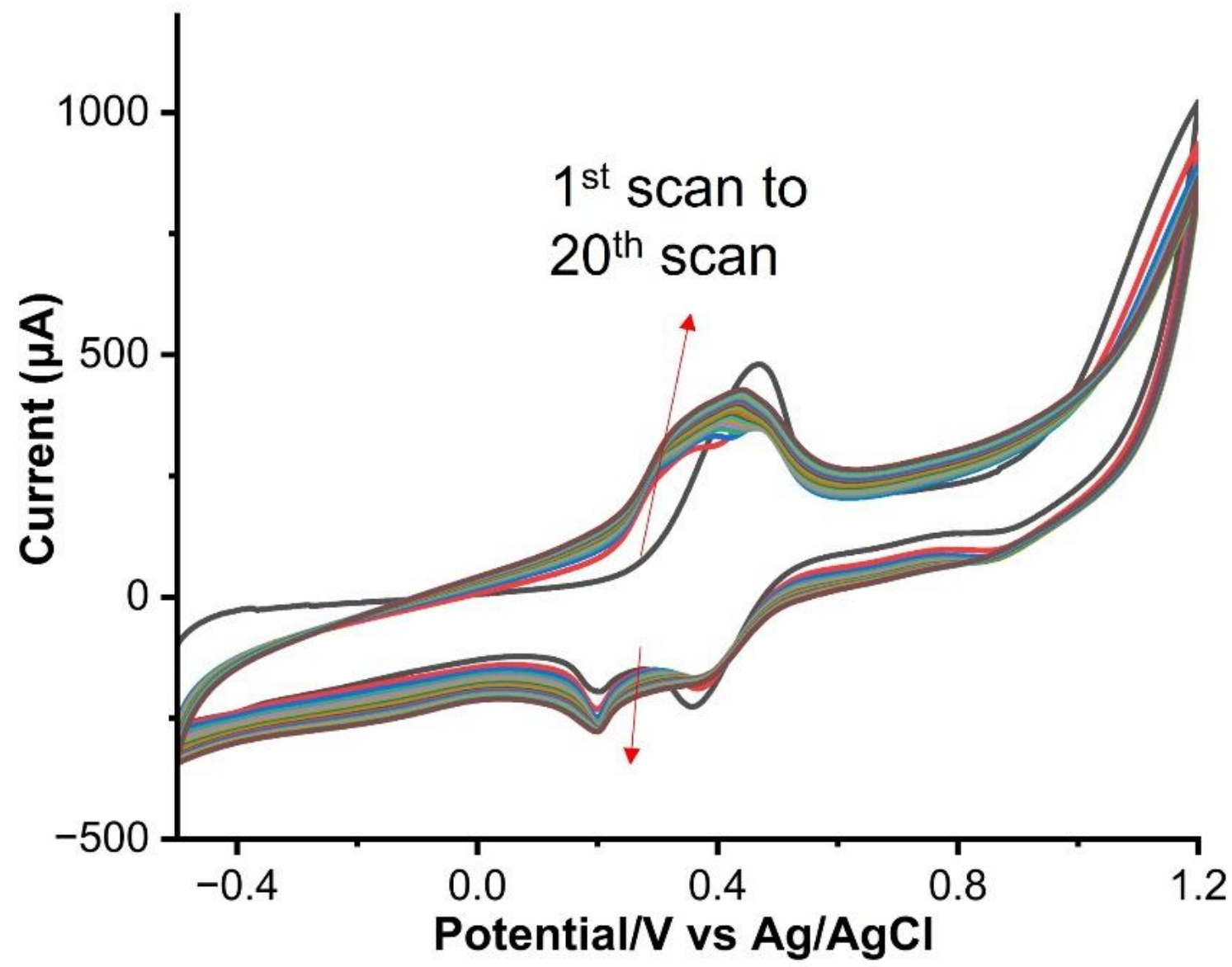

Figure S12. CVs of electrochemical deposition of PEDOT:PB on SLSG electrode over the range of -0.5~1.2 V in 0.1 M KCl and HCl containing 10 mM EDOT monomer and 10 mM mixture of $K_4[Fe(CN)_6]$ and $FeCl_2$ with the scan rate of 50 mV/s for 20 cycles.

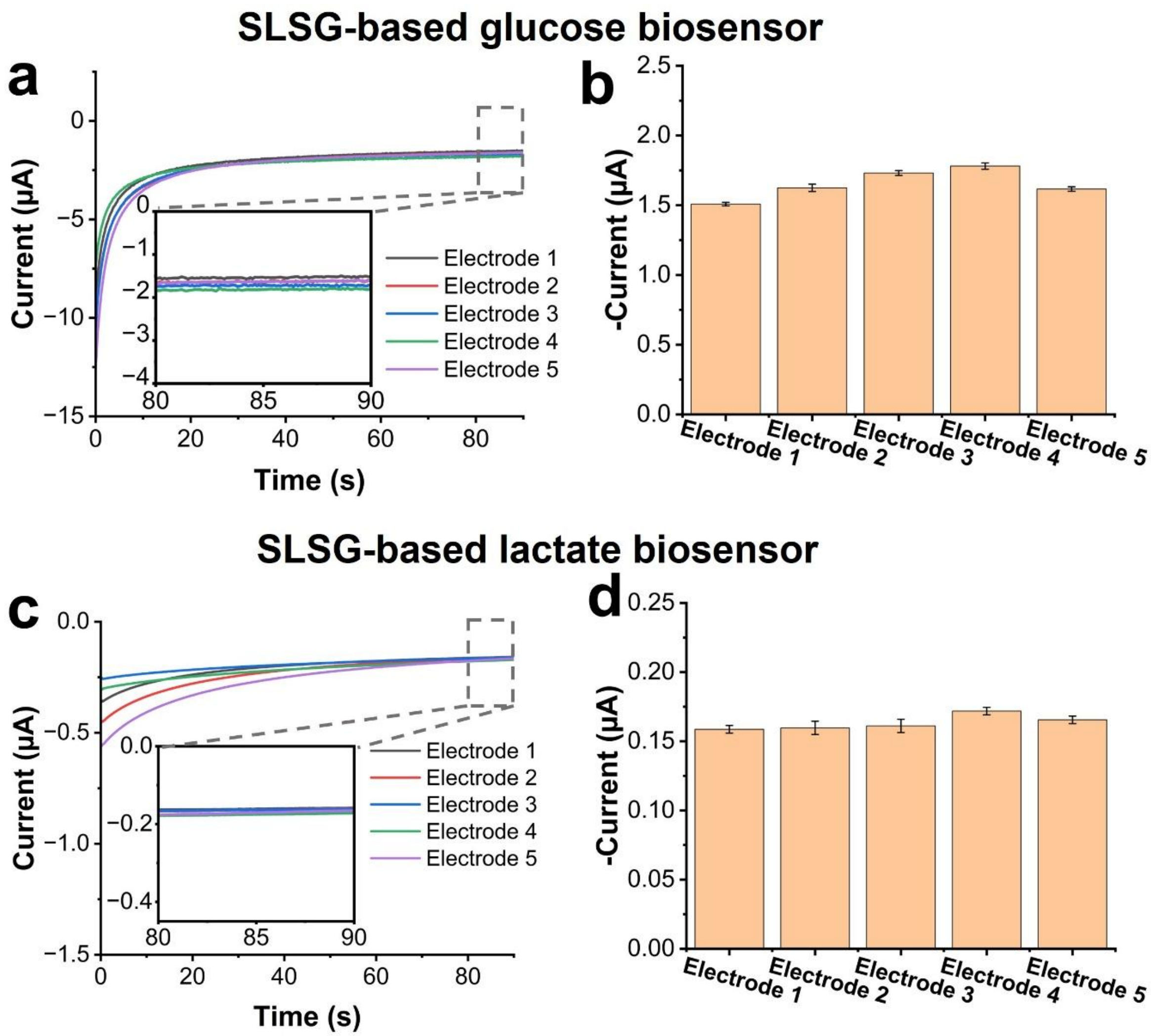

Figure S13. Reproducibility test of the glucose and lactate biosensor.

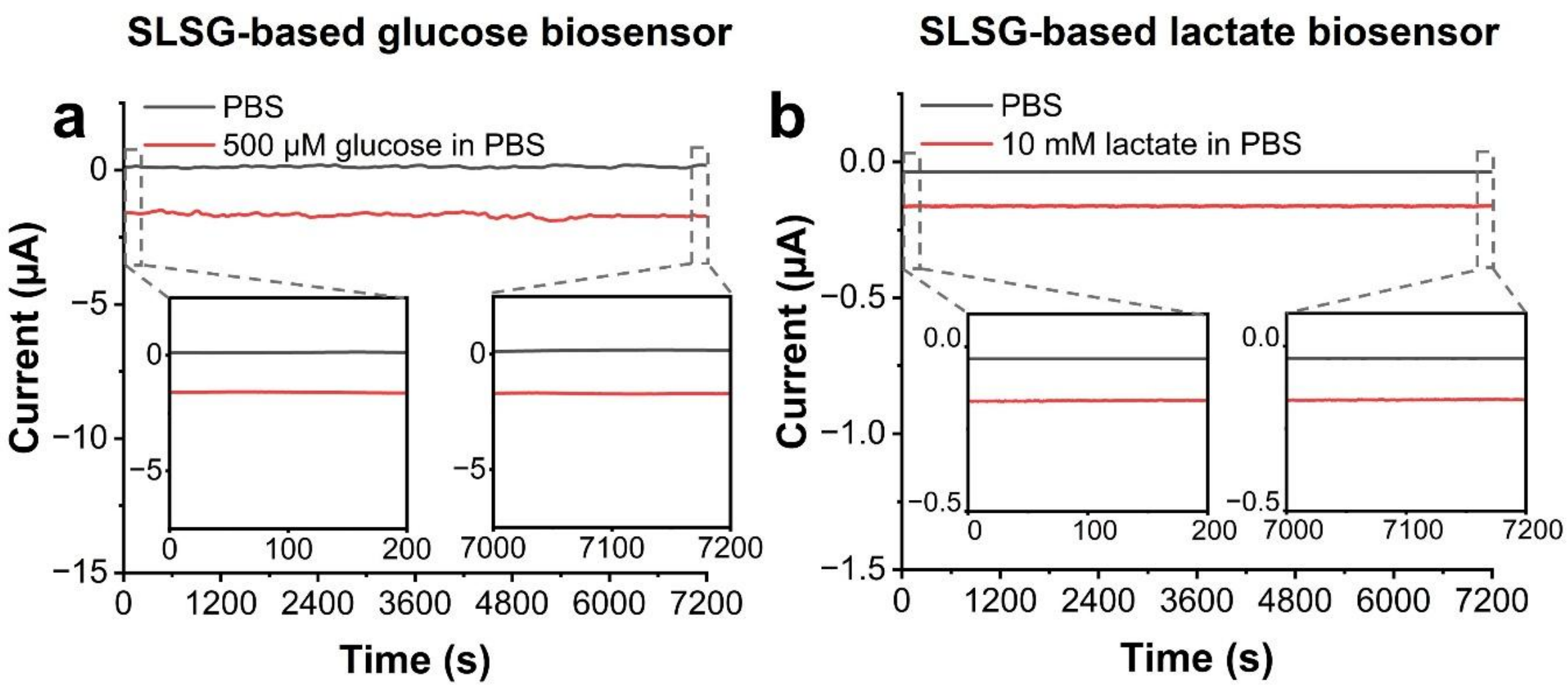

Figure S14. Operational long-term stability test of glucose and lactate biosensor.

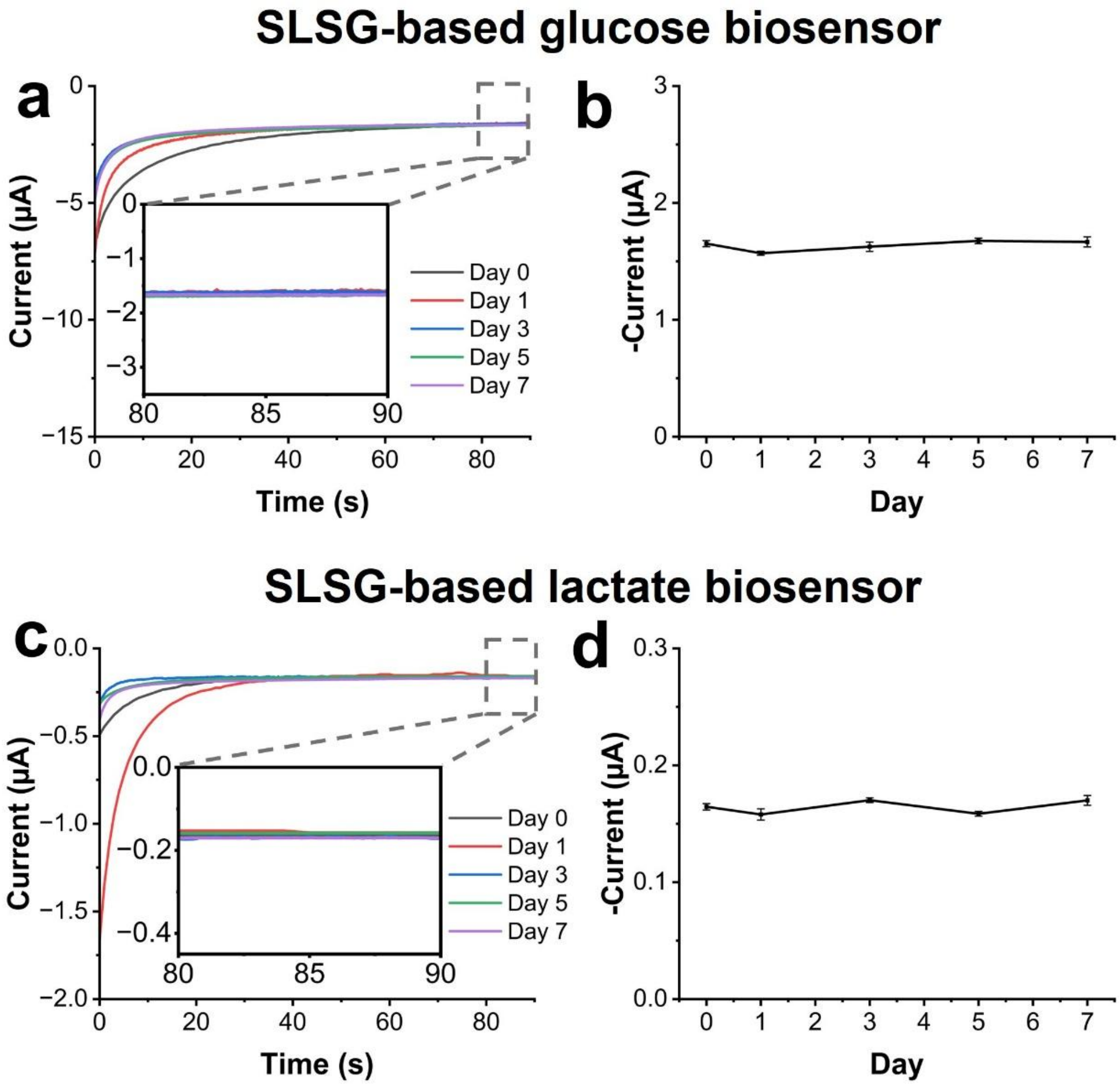

Figure S15. Storage long-term stability test of glucose and lactate biosensor.

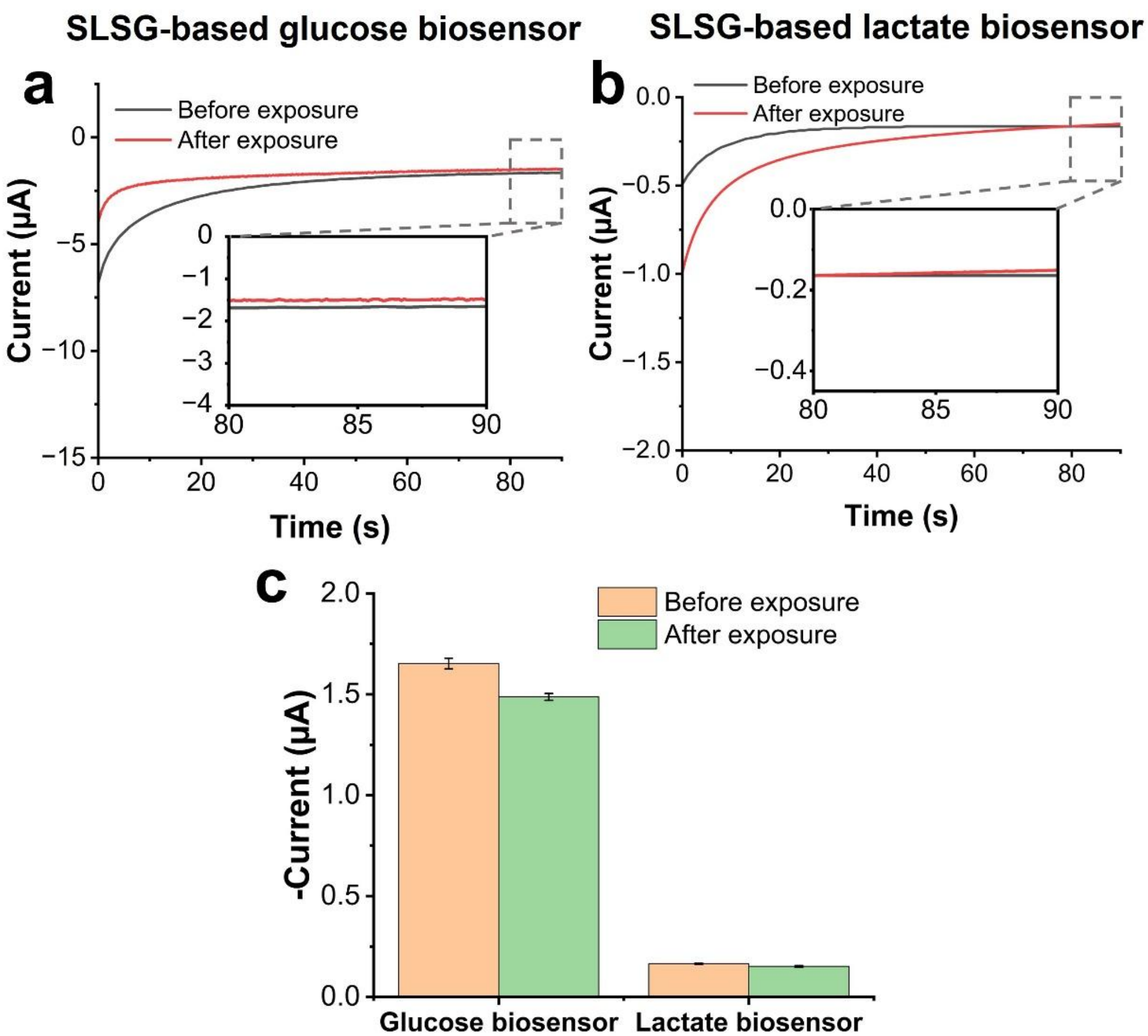

Figure S16. Anti-fouling test of glucose and lactate biosensor.

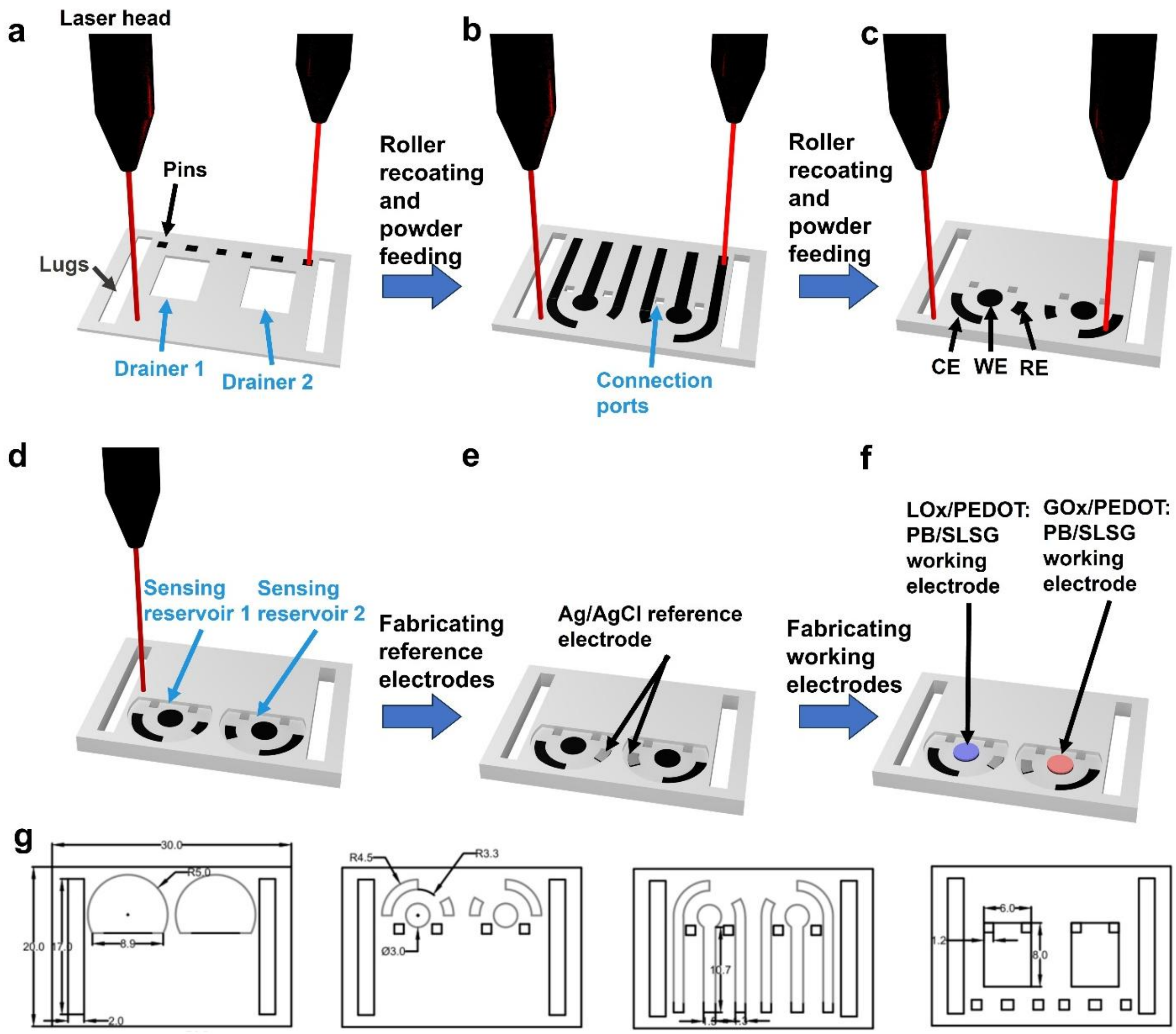

Figure S17. Schematic diagram of the fabrication process of the UMMB (a~f) and the unibody design layout of the UMMB (g), unit: mm.

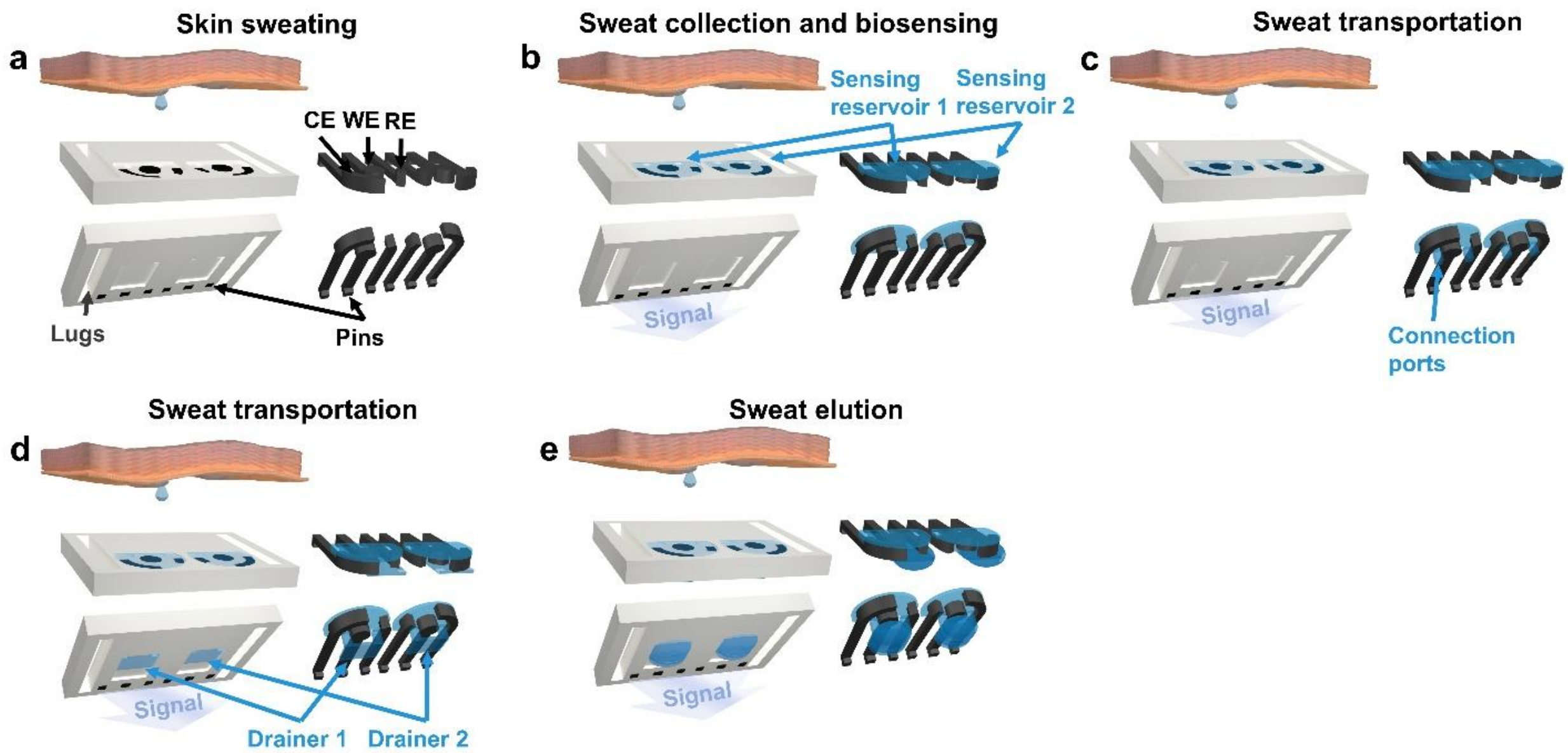

Figure S18. Schematic diagram of the working process of the UMMB.

Table S1. Systematic comparison of the SLSG technology with conventional fabrication technologies described in the literature.

Fabrication methods	Materials	Stereo-circuits	Stereo-fluidics	Post-integration / fabrication complexity	Instrument requirement	Manufacturing time and steps	Customization ability	Scalability / Yield	Cost
**SLSG (This work)**	PEEK (mono-matrix)	Yes	Yes	No, simultaneous fabrication	Laser, powder recoating machine	Single step; Simultaneous laser photothermal process	High; customization for every object	High; depending on size of laser chamber and roller	Low; material, electricity fee
**Photolithography / PDMS soft lithography + bonding**[8]	PDMS + metal electrodes	No	Yes	Yes; molding + bonding	UV mask aligner + cleanroom + mold tooling + plasma bonder	Multi-step	Low; new mask per design	Moderate; fabrication complexity-limited	High; material, cleanroom, masks, plasma fee
**Injection molding / hot embossing / casting**[9]	Thermoplastic (PMMA/PC) + metal electrodes	No	Yes	Yes; molding + assembly + sealing	Molding machine + mold tooling	Multi-step	Low; new mold and re-tooling per design	High at volume; low for prototyping	High; molding tooling fee
**Screen printing + separate fluidics + assembly**[10]	Conductive paste (carbon/Ag) + separate fluidic layer	No	Yes	Yes; printing + assembly	Screen printer + masks	Multi-step	Low; new mask per design	High at volume; low for prototyping; fabrication complexity-limited	Moderate; material, masks fee
**Inkjet printing + laser cutting + plasma-bonded PDMS**[11]	Conductive ink + PDMS	No	Yes	Yes; printing + cutting + assembly + bonding	Inkjet printer + laser cutter + plasma cleaner + cleanroom	Multi-step	Moderate; new assembly strategy per design	Moderate; fabrication complexity-limited	High; material, cleanroom, masks, plasma fee
**Laser direct writing + laser cutting + tape adhesion**[12,13]	Laser-induced graphene on polyimide + laser-cut adhesive film	No	Yes	Yes; writing + cutting + assembly + bonding	laser engraver + laser cutter	Multi-step	Moderate; new assembly strategy per design	Moderate; fabrication complexity-limited	Low; material, electricity fee
**Semisolid extrusion (SSE) + layer assembly**[14]	Extrudable conductive ink + elastomer	Yes	Yes	Yes; printing + material changing + assembly	Multi-nozzle extrusion 3D printer	Multi-step	Moderate; new assembly strategy per design	Moderate; fabrication complexity-limited	Moderate; specialized inks fee

Table S1. **Continued.** Systematic comparison of the SLSG technology with conventional fabrication technologies described in the literature.

**FDM multi-nozzle + embedded metallic interconnects**[15]	FDM thermoplastic + Ag-particle interconnects	Yes	No	Yes; printing + nozzle changing	Multi-nozzle FDM printer	Multi-step	Moderate; nozzle changing-constrained	Moderate; fabrication complexity-limited	High; specialized Ag inks fee
**Stereolithography (SLA) + direct ink writing (DIW)**[16]	Photopolymer resin + conductive (Ag) ink	Yes	No	Yes; printing + method changing + post-curing	SLA printer + DIW dispenser	Multi-step	Moderate; method changing-constrained	Moderate; fabrication complexity-limited	High; two systems, specialized Ag inks fee
**Dual-light curing / DLP (PEGDA + $AgNO_3$)**[17]	PEGDA + $AgNO_3$	Yes	No	Yes; printing + laser changing + post-curing	Dual-wavelength DLP printer	Multi-step	High	Moderate; fabrication complexity-limited	High; two lasers, specialized Ag inks fee

Note: Qualitative ratings (Low / Moderate / High) denote relative comparison synthesized from the cited literature and the present work.

Table S2. Quantitative Raman comparison.

	I_D/I_G	L_a	I_{2D}/I_G
SLSG sintered object	--	--	--
SLSG graphitized object	0.23	87.59	0.30
Graphitized polyimide[18]	1.38	13.93	0.48

Table S3. Comparison with other PB-based electrodes for sensing H_2O_2 described in the literature.

**Electrode**	**Sensitivity/ (μA/mM)**	**Linear range/ μM**	**LOD/ μM**	**Reference**
PB-PEDOT/SLSG-PEEK	62.54	5-1140	2.3	This work
PB-PEDOT/LIG	24.33	10-1760	2.5	[19]
PEDOT/PB/GCE	16.67	0.5-839	0.16	[20]
RGO/PB/GCE	51.26	0.8-500	0.25	[21]
CB-PB-Nafion/SPCE	0.66 μA/(mM*cm^2)	0.2-1000	1	[22]
PB/PANI/GO/GCE	60.16	0.5-1275	1.9	[23]

Table S4. Comparison with other LOx-based electrodes for sensing lactate described in the literature. “N.A.” means not available.

**Electrode**	**Sensitivity (µA/mM)**	**Linear range (mM)***	**LOD (µM)**	**Reference**
LOx/PB-PEDOT/SLSG-PEEK	29.43	4-20	0.3	This work
Integrated LOx/PB-PEDOT/LIG	2.23	0-18	6.8	[19]
LOx-PPD/PB-SPCE	0.553	0.1-1.0	50	[24]
LOx-PPD/HRP-MCNTs-PEGDGE/SPCE	0.74	0-25	40	[25]
Chit-LOx-BSA/ PB-SPCE	0.096	0-28	N.A.	[26]
LOx/PB/SPCE-superhydrophobic carbon	3.56	0-30	5	[27]

Note: Physiological range of lactate in human sweat is ~4-20 mM.[28]

Table S5. Comparison with other GOx-based electrodes for sensing glucose described in the literature.

Electrode	Sensitivity (μA/mM)	Linear range (mM)*	LOD (μM)	Reference
GOx/PB-PEDOT/SLSG-PEEK	16.86	0.01-1	3	This work
GOx/PB/graphene-hybrid	1.0	0.01 – 0.7	< 10	[29]
GOx-PB/N-doped carbon/GCE	1.67	0.005-5	1	[30]
GOx/PtNP/acetic acid treated LIG	4.62	0.0003 – 2.1	0.3	[31]
GOx/Chitosan-modified LIG	3.05	0–8	431	[32]
CPE/GOx-SiO_2/LIG	0.78	0.5–9	145	[33]

Note: Physiological range of glucose in human sweat is ~0.02–0.6 mM.[34]

Table S6. Comparison of the long-term stability, anti-fouling properties, and reproducibility with representative bioelectrodes/biosensors reported in the literature.

Bioelectrode / biosensor	Target analyte	Reproducibility	Operational long-term stability	Storage long-term stability	Anti-fouling / biofluid robustness	Reference
**GOx/PB-PEDOT/SLSG-PEEK**	Glucose	Inter-CV = 6.5%; intra-CV < 1.6%	93.7% after 2 h in 500 μM glucose	CV = 2.6%; >92.9% after 7 days	90% after 2 h real sweat exposure	This work
**LOx/PB-PEDOT/SLSG-PEEK**	Lactate	Inter-CV = 3.3%; intra-CV < 3.0%	98.1% after 2 h in 10 mM lactate	CV = 3.6%; >95.0% after 7 days	92% after 2 h real sweat exposure	This work
**GOx–chitosan/TiO_2 nanotube arrays**	Glucose	Inter-CV = 2.5%; intra-CV = 1.9%	N.A.	85% after 30 days	N.A.	[35]
**LOx/N-doped CNT electrode**	Lactate	Inter-CV = 5.0%; intra-CV = 1.6%	N.A.	Repeatability CV < 3.4% over 90 days	N.A.	[36]
**LOx/PtNPs/GCNF/SPCE**	Lactate	Inter-CV = 4.9%; intra-CV = 1.8%	90% after 50 measurements	90% after 3 months (RT); 95% after 18 months (−20 °C)	N.A.	[37]
**LDH/GOx/PEDOT:PSS OECT**	Glucose	Intraday slope variation < 5%	N.A.	100% after 5 days; 70% after 10 days	With Nafion: UA, no detectable change; AA, +7.0 ± 0.8%	[38]
**ADH/pMB-MWCNTs/SPG and AOx/hemin-MWCNTs/SPG**	Ethanol	CV = 8.1%	>2800 s continuous turnover (self-powered)	~93% after 90 days	N.A.	[39]
**HT–poly-A/Au electrode**	Exosomal miR-21	CV < 4.5%	N.A.	97.26% after 7 days	63.16% after 1 h in 50% human serum	[40]

Note: N.A. means not available or not explicitly reported.

References

[1] A. P. Schuetze, W. Lewis, C. Brown, W. J. Geerts, *Am. J. Phys.* **2004**, *72*, 149.
[2] T. Larsen, T. Larsen, S. J. Andreasen, J. D. C. Christiansen, *Int. J. Hydrog. Energy* **2023**, *48*, 12493.
[3] F. T. Arecchi, *Phys. Rev. Lett.* **1965**, *15*, 912.
[4] J. T. Luxon, D. E. Parker, *Appl. Opt.* **1981**, *20*, 1933.
[5] L. G. Cançado, K. Takai, T. Enoki, M. Endo, Y. A. Kim, H. Mizusaki, A. Jorio, L. N. Coelho, R. Magalhães-Paniago, M. A. Pimenta, *Appl. Phys. Lett.* **2006**, *88*, 163106.
[6] M. Karpacheva, C. E. Housecroft, E. C. Constable, *Beilstein J. Nanotechnol.* **2018**, *9*, 3069.
[7] R. Shi, L. Zhang, T. Gao, W. Lian, K. Liu, X. Zhao, B. Li, N. Chen, W. Song, *Dalton Trans.* **2022**, *51*, 14201.
[8] J. Ma, L. Jiang, X. Pan, H. Ma, B. Lin, J. Qin, *Microfluid. Nanofluid* **2010**, *9*, 1247.
[9] N. Convery, I. Samardzhieva, J. M. Stormonth-Darling, S. Harrison, G. J. Sullivan, N. Gadegaard, *Macromol. Mater. Eng.* **2021**, *306*, 2100464.
[10] G. Paimard, E. Ghasali, M. Baeza, *Chemosensors* **2023**, *11*, 113.
[11] C. Xu, Y. Song, J. R. Sempionatto, S. A. Solomon, Y. Yu, H. Y. Y. Nyein, R. Y. Tay, J. Li, W. Heng, J. Min, A. Lao, T. K. Hsiai, J. A. Sumner, W. Gao, *Nat. Electron.* **2024**, *7*, 168.
[12] Y. Yang, Y. Song, X. Bo, J. Min, O. S. Pak, L. Zhu, M. Wang, J. Tu, A. Kogan, H. Zhang, T. K. Hsiai, Z. Li, W. Gao, *Nat. Biotechnol.* **2020**, *38*, 217.
[13] R. Ye, D. K. James, J. M. Tour, *Adv. Mater.* **2019**, *31*, 1803621.
[14] Y. Song, R. Y. Tay, J. Li, C. Xu, J. Min, E. Shirzaei Sani, G. Kim, W. Heng, I. Kim, W. Gao, *Sci. Adv.* **2023**, *9*, eadi6492.
[15] S.-Y. Wu, C. Yang, W. Hsu, L. Lin, *Microsyst. Nanoeng.* **2015**, *1*, 15013.
[16] A. Joe Lopes, E. MacDonald, R. B. Wicker, *Rapid Prototyp. J.* **2012**, *18*, 129.
[17] J. Xiao, D. Zhang, Q. Guo, J. Yang, *Adv. Mater. Technol.* **2021**, *6*, 2100039.
[18] G. F. Hawes, D. Yilman, B. S. Noremberg, M. A. Pope, *ACS Appl. Nano Mater.* **2019**, *2*, 6312.
[19] L. Meng, A. P. F. Turner, W. C. Mak, *ACS Appl. Mater. Interfaces* **2021**, *13*, 54456.
[20] J. Wang, Y. Wang, M. Cui, S. Xu, X. Luo, *Microchim. Acta* **2017**, *184*, 483.
[21] J. Li, Y. Jiang, Y. Zhai, H. Liu, L. Li, *Anal. Lett.* **2015**, *48*, 2786.
[22] D. Rojas, F. Della Pelle, M. Del Carlo, M. d'Angelo, R. Dominguez-Benot, A. Cimini, A. Escarpa, D. Compagnone, *Sens. Actuators B Chem.* **2018**, *275*, 402.
[23] Z. Yang, X. Zheng, J. Zheng, *Synth. Met.* **2016**, *221*, 153.
[24] J. Kim, G. Valdés-Ramírez, A. J. Bandodkar, W. Jia, A. G. Martinez, J. Ramírez, P. Mercier, J. Wang, *Analyst* **2014**, *139*, 1632.
[25] L. Zhang, J. Liu, Z. Fu, L. Qi, *J. Nanosci. Nanotechnol.* **2020**, *20*, 1495.
[26] S. Imani, A. J. Bandodkar, A. M. V. Mohan, R. Kumar, S. Yu, J. Wang, P. P. Mercier, *Nat. Commun.* **2016**, *7*, 11650.
[27] L. Huang, M. Zeng, Y. Li, Z. Liu, J. Zhang, X. Feng, *ACS Sens.* **2025**, *10*, 3433.
[28] G. Yang, J. Hong, S.-B. Park, *Front. Physiol.* **2024**, *15*, 1376801.
[29] H. Lee, T. K. Choi, Y. B. Lee, H. R. Cho, R. Ghaffari, L. Wang, H. J. Choi, T. D. Chung, N. Lu, T. Hyeon, S. H. Choi, D.-H. Kim, *Nat. Nanotechnol.* **2016**, *11*, 566.
[30] A. F. Quintero-Jaime, J. Quílez-Bermejo, D. Cazorla-Amorós, E. Morallón, *Electrochim Acta* **2021**, *367*, 137434.

[31] H. Yoon, J. Nah, H. Kim, S. Ko, M. Sharifuzzaman, S. C. Barman, X. Xuan, J. Kim, J. Y. Park, *Sens. Actuators B Chem.* **2020**, *311*, 127866.
[32] K. Settu, P.-T. Chiu, Y.-M. Huang, *Polymers* **2021**, *13*, 2795.
[33] A. Jędrzak, T. Rębiś, Ł. Klapiszewski, J. Zdarta, G. Milczarek, T. Jesionowski, *Sens. Actuators B Chem.* **2018**, *256*, 176.
[34] A. Y. Alhaddad, H. Aly, H. Gad, A. Al-Ali, K. K. Sadasivuni, J.-J. Cabibihan, R. A. Malik, *Front. Bioeng. Biotechnol.* **2022**, *10*, 876672.
[35] M. Artigues, J. Abellà, S. Colominas, *Sensors* **2017**, *17*, 2620.
[36] J. M. Goran, J. L. Lyon, K. J. Stevenson, *Anal. Chem.* **2011**, *83*, 8123.
[37] O. A. Loaiza, P. J. Lamas-Ardisana, L. Añorga, E. Jubete, V. Ruiz, M. Borghei, G. Cabañero, H. J. Grande, *Bioelectrochemistry* **2015**, *101*, 58.
[38] I. Gualandi, M. Tessarolo, F. Mariani, D. Arcangeli, L. Possanzini, D. Tonelli, B. Fraboni, E. Scavetta, *Sensors* **2020**, *20*, 3453.
[39] V. Marchianò, A. Tricase, E. Macchia, P. Bollella, L. Torsi, *Anal. Bioanal. Chem.* **2024**, *416*, 5303.
[40] G. Chen, W. Chen, L. Xu, H. Jin, W. Sun, J. Lan, F. Wu, X. Zhang, J. Zhang, J. Chen, *Anal. Chem.* **2022**, *94*, 5382.
